# Accuracy in the prediction of disease epidemics when ensembling simple but highly correlated models

**DOI:** 10.1371/journal.pcbi.1008831

**Published:** 2021-03-15

**Authors:** Denis A. Shah, Erick D. De Wolf, Pierce A. Paul, Laurence V. Madden

**Affiliations:** 1 Department of Plant Pathology, Kansas State University, Manhattan, Kansas, United States of America; 2 Department of Plant Pathology, The Ohio State University, Ohio Agricultural Research and Development Center, Wooster, Ohio, United States of America; University of Cambridge, UNITED KINGDOM

## Abstract

Ensembling combines the predictions made by individual component base models with the goal of achieving a predictive accuracy that is better than that of any one of the constituent member models. Diversity among the base models in terms of predictions is a crucial criterion in ensembling. However, there are practical instances when the available base models produce highly correlated predictions, because they may have been developed within the same research group or may have been built from the same underlying algorithm. We investigated, via a case study on Fusarium head blight (FHB) on wheat in the U.S., whether ensembles of simple yet highly correlated models for predicting the risk of FHB epidemics, all generated from logistic regression, provided any benefit to predictive performance, despite relatively low levels of base model diversity. Three ensembling methods were explored: soft voting, weighted averaging of smaller subsets of the base models, and penalized regression as a stacking algorithm. Soft voting and weighted model averages were generally better at classification than the base models, though not universally so. The performances of stacked regressions were superior to those of the other two ensembling methods we analyzed in this study. Ensembling simple yet correlated models is computationally feasible and is therefore worth pursuing for models of epidemic risk.

## Author summary

Ensembling takes a set of predictions from individual models and combines them such that the performance of the ensemble is ideally better than that of any one of the constituent models. Ensembling requires diversity among the individual models in terms of their predictions. However, models developed within the same research group may in fact be interrelated, and high levels of correlation among their predictions could theoretically negate any ensembling benefit. We examined, using a case study on predicting epidemics of Fusarium head blight of wheat, whether ensembling could still be beneficial when the individual models were simple but highly correlated. Even in this situation ensembling led to improvements in prediction without a high computational cost and was therefore profitable even when the diversity in model predictions was low.

## Introduction

When making important decisions, we naturally seek different opinions. Translated to prediction, this means consulting different models, each of which makes predictions with a level of uncertainty, inasmuch that any model only approximates the truth. Combining, or ensembling, the predictions made by several individual models can lead to a prediction that is overall better and more stable (less variable) than those given by any one of the component models [1,2]. That is, ensembling uses multiple models to reduce the risk of incorrect predictions and improve forecasts [3]. Model ensembling has been investigated since the 1970s [1], but has seen deeper exploration in infectious disease epidemiology only recently [4–6] and is just beginning to appear in botanical epidemiology [7–9], a field that has a tradition of statistically selecting one ‘optimal’ model.

The individual models (base learners) in an ensemble ideally should exhibit low correlations when their predictions are compared [1,10], as this enables the higher-level ensembling algorithm (the meta-learner) to find a combination of those predictions that improves upon the prediction made by any one base learner model. Put another way, ensembling requires the base learners to make different errors on the observations [10]. In effect, the risk of accepting a poor prediction made by a model for a given observation is reduced. The assumption is that base learners are skillful in different ways, performing better on some observations than on others. If the base learners are highly correlated (i.e., make very similar predictions on the same observations) then the theory suggests that the benefits of ensembling are negated.

However, model building is an interdependent process. Disease models can be influenced by common theory underlying processes driving disease development, groups working on the same or similar diseases may share code and ideas, and models worked on within a single research group may evolve progressively over time as modifications and improvements are incorporated. Hence, models coming from a single group tend to be related within and across model generations [11,12]; and correlations among models from different research groups can develop as researchers weigh or include results from others. The interrelatedness among models over the period of their development can be called a model genealogy [12]. Model simplicity imposes some diversity by inciting a level of error in the predictions, but the correlations among the predictions work against diversity among the base learners. In this paper, we explored whether any benefit can be derived from ensembling simple yet highly correlated models for predicting the risk of a plant disease epidemic.

For the case study, we fit ensembles of simple logistic regression models used to predict the risk of epidemics (and non-epidemics) of Fusarium head blight (FHB) in U.S. wheat. The disease level in wheat fields is classified as epidemic or non-epidemic based on the magnitude of the predicted risk probability. Our research group has been working on these types of models over the past 17 years [7,13–16]. The logistic regression models are now at the third generation, several of which have not yet been published and will become part of this paper. FHB is a fungal disease caused by members of the *Fusarium graminearum* species complex [17] and is one of the most economically concerning diseases of wheat globally; not only because of yield reduction but especially because of the production of mammalian toxins such as deoxynivalenol in the wheat grain [18]. Applying a protectant fungicide during wheat flowering (anthesis) is one of the main ways of controlling the disease, thereby reducing the risk of mycotoxin contamination [19]. The fungicide application is however not needed every year or in all locations [20]. The risk of disease, and hence the need for fungicide, is high only when environmental (mainly weather) conditions are favorable [16]. Our models attempt to predict when and where those favorable conditions translate into disease epidemics.

The models are the basis of daily updated risk maps covering over 30 U.S. States during the wheat growing season (http://www.wheatscab.psu.edu/). The spatiotemporal scale and rapid update cycle at which the models are run, and at which results are projected, are the main reasons we have focused on the logistic regression algorithm, because of low computational cost and scalability [3]. An ensembling algorithm would have to meet these criteria as well. Other important criteria were model simplicity and interpretability to respect and serve the needs of a wheat-producer-oriented clientele [21]. Profit margins are slim in U.S. wheat production. When grain prices are low, spending money on a fungicide without realizing a return on the investment (improved yield or grain quality) could mean a net loss for the grower. Accuracy in predicting epidemics is a given; a false positive prediction means that a grower could unnecessarily spray a field with fungicide, whereas a false negative could mean, besides yield reductions, price discounts or complete grain rejection due to unacceptably high levels of mycotoxin contamination [22].

The two main methods for combining base learners fall under weighting and meta-learning [1]. Stacking, a popular approach within meta-learning, is typically used to combine models built using different algorithms (or inducers, in the language of [1]; an inducer is the algorithm that is used to construct the model and the fitted model is the predictor or classifier, in this case the FHB epidemic model). With the FHB case study, all base learner models were derived from the same algorithm (logistic regression). Ensembling models stemming from the same inducer would upon first inspection violate the diversity principle discussed above. However, diversity among models can be generated in other ways. One popular approach is feature set partitioning [1]. The original set of available predictor variables is divided into several smaller (possibly overlapping) subsets, each of which is then used to train a model. The benefits of feature set partitioning are a decrease in computational complexity; as the models are smaller, higher interpretability is possible. Feature set partitioning therefore fits within our operational criteria for large-scale deployment of FHB predictive models. The FHB logistic regression models consisted of no more than four weather-based predictor variables [15] out of a full set of about 300 candidate weather-based predictor variables, though some overlap in the use of predictors by models was allowed (i.e., soft boundaries on the partitioned feature space).

The objective was to investigate three model ensembling techniques (soft voting, weighted averaging, and stacking) for their ability to improve model performance relative to that of base learners, under the condition that the base learners were simple models all induced from the same algorithm and with the further property of high correlations among their predicted probabilities. Throughout the paper we refer to the models as base learners (within the context of ensembling) or as individual logistic regression models. Applying the methods to the FHB case study showed that although the individual logistic regression models had correlated predicted probabilities, they could be successfully ensembled, with penalized stacking providing the most benefit.

## Results

### Fitted probabilities and classifications for individual (base learner) models

The 39 logistic regression models were positively correlated, and in many cases highly so, in terms of their cross-validated (cv) probabilities of epidemics across the observations. The Pearson correlation between these probabilities for any pair of models was 0.782, on average. The minimum such correlation was 0.577 and the maximum was 0.996. There were clearly groups of models with very similar cv probabilities (correlations above 0.9, for instance) and other groups with less agreement in their predictions (correlations below 0.7); see S1 Fig. There was a distribution in the cv probabilities of epidemics returned by the 39 models for any given observation (illustrated for a sample of observations in S2 Fig). On a single observation basis, less variability in the predicted probabilities was seen with observations from wheat varieties in the moderately susceptible and the moderately resistant classes; these two resistance classes generally show lower levels of disease severity in the field (than the other two classes) and hence lower frequencies of observed epidemics. This variability in predicted probabilities translated to some diversity in model classification of epidemics and non-epidemics after the conversion of probabilities to a predicted class membership. Some observations were perfectly classified by all logistic regression models, others were misclassified by every single model, and other observations were correctly classified to varying degrees of success (as shown in S3 Fig). None of the 39 models were identical in terms of the classifications they returned over the entire set of observations.

### Brier scores

Models were highly correlated in terms of their Brier scores (*B*_*m*,*i*_) calculated on the cv probabilities of epidemics returned by each model *m* (*m* = 1, …,39) for each observation *i* (S4 Fig). Taking any pair of logistic regression models *m* and m* (*m* ≠ m*), the average Pearson correlation between *B*_*m*,*i*_ and $B_{m^{*},i}$ was 0.867; the minimum and maximum pairwise correlations were 0.683 and 0.997, respectively. The mean Brier scores of the models, ${\bar{B}}_{m}$, varied from a minimum of 0.160 to a maximum of 0.183 (average of 0.171, standard error of the mean = 0.00078) with model M3 the obvious outlier (${\bar{B}}_{3}$ = 0.183; see S5 Fig). For context, a perfect ${\bar{B}}_{m}$ score is 0, and the worst possible ${\bar{B}}_{m}$ score is 1. The outlier model M3 was not included in the ensembles.

### Model genealogy

**Fig 1:**
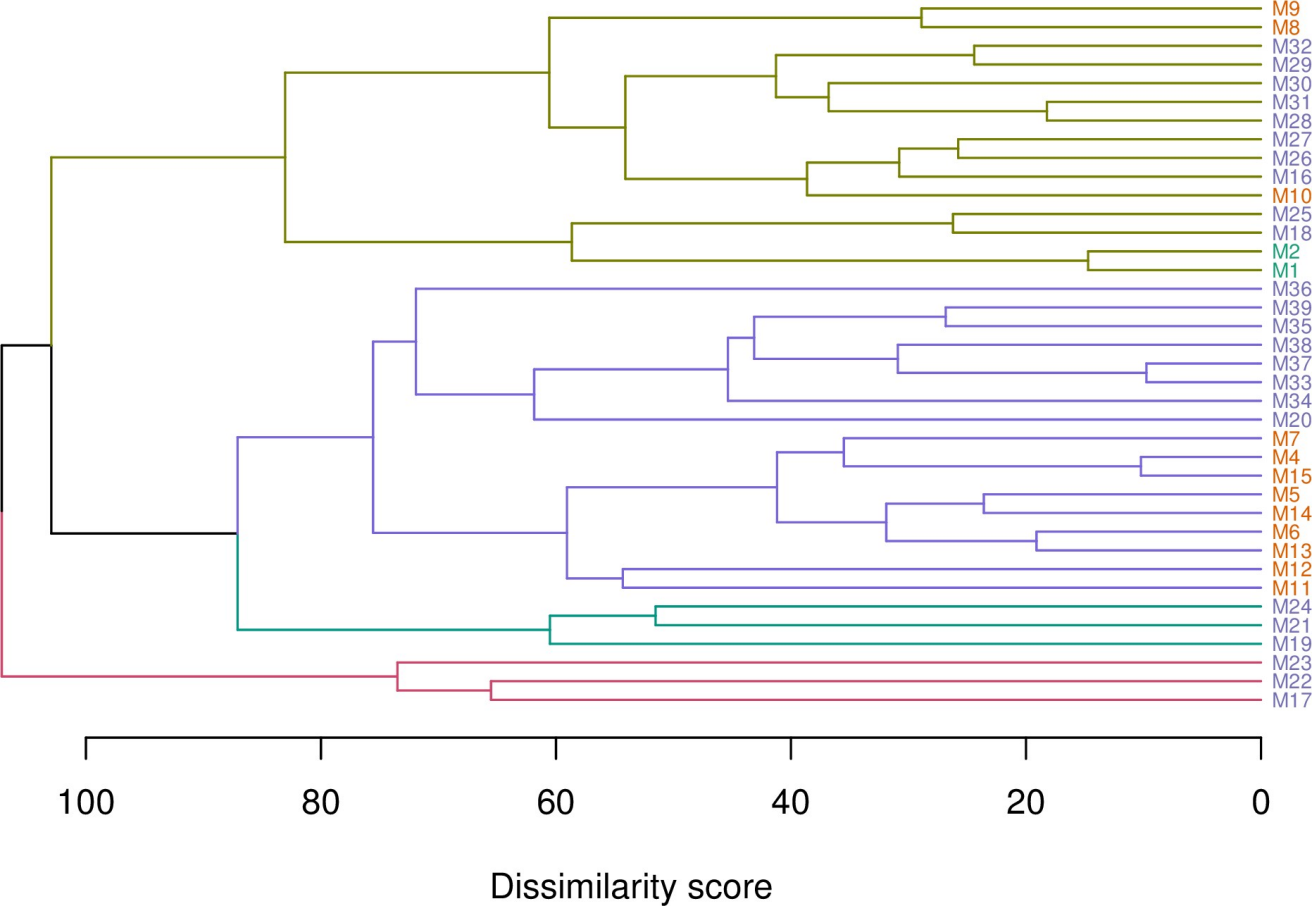
Hierarchical clustering of the logistic regression models. Clustering of models based on the Brier scores using the Manhattan distance metric estimated from a 999 x 38 data matrix of *B*_*m*,*i*_ values. Grouping was done using the ‘complete’ agglomeration method on the distance matrix. Labels are colored by model generation: green, 1^st^ generation; orange, 2^nd^ generation; purple, 3^rd^ generation. Four groups of models are indicated by the branch colors.

A ‘family tree’ dendrogram based on the model Brier scores captured the genealogical evolution of the logistic regression models (Fig 1). Four groups of models were identified. The two earliest-developed models M1 and M2 [13] clustered together. All but three of the 2^nd^ generation models [7,15] were grouped together. The 3^rd^ generation models were more scattered, being found in all four groups, reflecting a greater diversity in this generation of models.

### Model ensembles

Soft voting simply averaged the unweighted cv probabilities of epidemics across all the base learners. The weighted model averaging approach made use of the family dendrogram (Fig 1) to partition the base learners into four groups. Selecting one model per partition led to a subset of four models, whose (mean Brier score) weighted cv probabilities were then averaged. This process was repeated for a random selection of 10 subsets, out of the total number of possible permutations given the partitions, resulting in 10 model-averaged models *M*_*x*_, *x* = 1, …, 10. The third ensembling approach (stacking) fit meta-learner penalized logistic regressions to the cv probabilities of epidemics returned by each of the 38 base learners (with epidemic status as the binary response). The fitted meta-learner models were used to predict the probability of epidemics.

**Fig 2:**
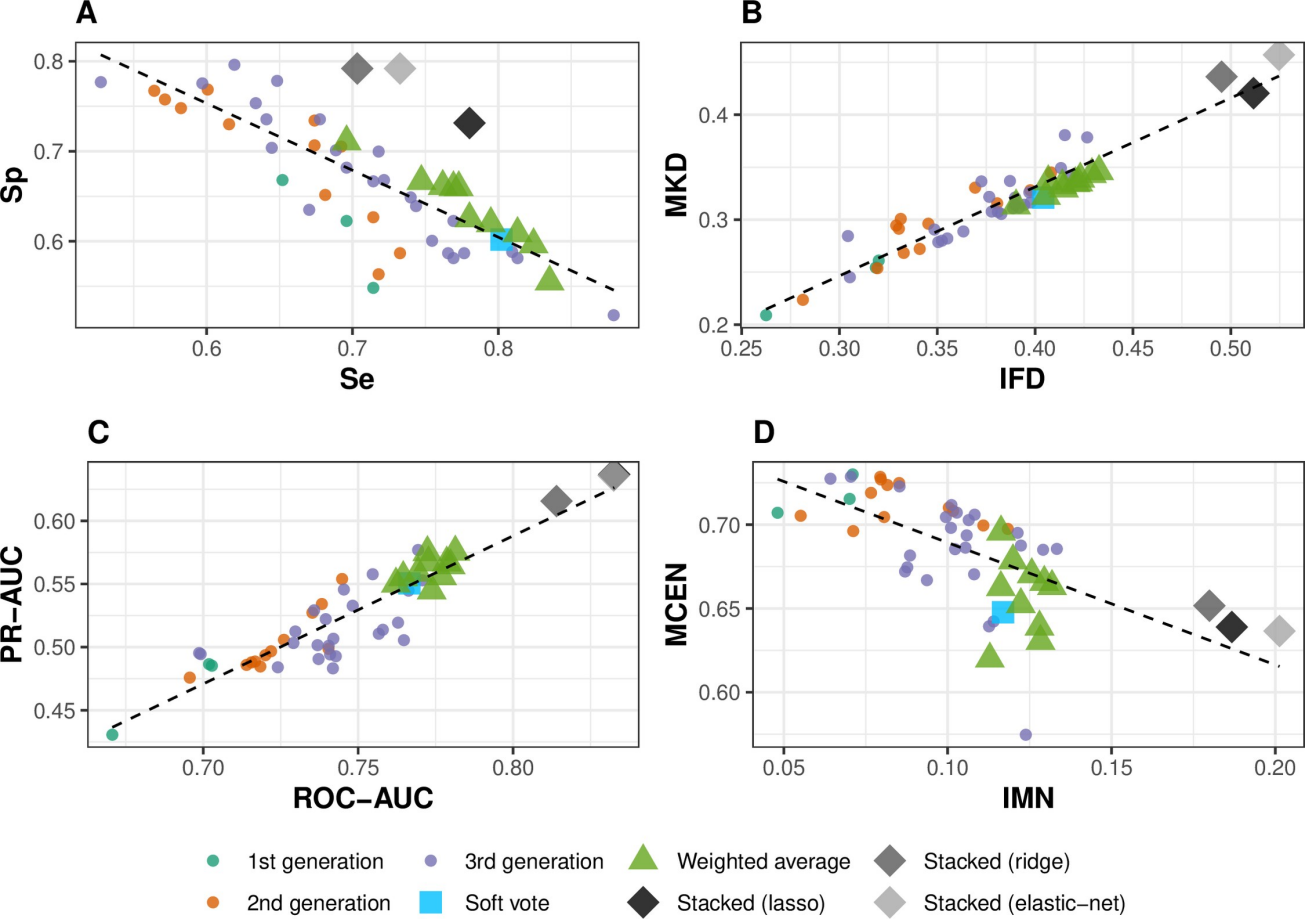
Performance of 38 base learner logistic models (identified by the generation of model building), and several ensembles. The ensembles are: a simple soft-vote model average across all base learner models; 10 weighted averages (*M*_*x*_) of four base learner models (where the sets of four were randomly chosen from the larger set of all possible permutations of selecting one model each from the four groups indicated in Fig 1, weights based on Brier scores); stacked regression models (with lasso, ridge or elastic-net penalizations) fitted to the cross-validated probabilities of epidemics from all base learner models. A. specificity (Sp) versus sensitivity (Se); B. markedness (MKD) versus informedness (IFD); C. area under the precision-recall curve (PR-AUC) versus the area under the receiver operating characteristic curve (ROC-AUC); D. modified confusion entropy (MCEN) versus the normalized expected mutual information (IMN). The dashed line in each panel is a linear regression through the data and serves as a referential aid. Metrics are defined in Table 1.

The presentation that follows is conditional on using the cut-points that maximized the Youden Index (YI = Se + Sp – 1, where Se is sensitivity and Sp is specificity; Table 1) for the respective models. That is, the cut-point was estimated separately for each model. Fig 2A shows a trade-off between Sp and Se, which we have observed before [15], apparent not only with the base learners but with the *M*_*x*_ models as well. Note that this Se–Sp trade-off is different from that observed by varying the cut-point on the probabilities returned by a single model. The stacked regression model with a ridge penalty favored Sp over Se, whereas using a lasso penalty favored Se over Sp. Elastic-net Se and Sp were in between those of the other two penalized models. Fig 2 also shows one other metric pair representing cut-point-dependent metrics, namely markedness (MKD = positive predictive value + negative predictive value - 1) versus informedness (IFD [23]; same as YI algebraically, but derived from different principles), one pair of ranking (cut-point independent) metrics [the area under the precision-recall curve (PR-AUC) versus the area under the ROC curve (ROC-AUC)] and one pair of information-theoretic or entropy-based metrics [the modified confusion entropy (MCEN) versus the normalized expected mutual information (IMN)]. The MCEN metric is a measure of classification-generated uncertainty (lower is a better score) and IMN ranges from 0 (a model is completely incapable of predicting epidemics) to 1 (a model predicts epidemics perfectly). The plots in Fig 2 indicate a degree of separation, at least visually, between the 38 base learners and the different ensembles.

**Table 1:** Definitions of terms associated with the confusion matrix and descriptions of binary classification metrics.

Symbol	Description and other names	Formula
**Confusion matrix terms**		
TP	True positive count	
FP	False positive count	
TN	True negative count	
FN	False negative count	
N	Total number of observations	TP + TN + FP + FN
AP	All actual positives	TP + FN
AN	All actual negatives	TN + FP
PP	All predicted positives	FP + TP
PN	All predicted negatives	FN + TN
TPn	Normalized true positives	TP/N
FPn	Normalized false positives	FP/N
TNn	Normalized true negatives	TN/N
FNn	Normalized false negatives	FN/N
**Sensitivity-specificity-type metrics**		
Se	Sensitivity = true positive rate = recall	TP/AP
Sp	Specificity = true negative rate = inverse recall	TN/AN
IFD	Informedness = Youden index	Se + Sp– 1
**PPV-NPV-type metrics**		
PPV	Positive predictive value = precision	TP/(TP + FP)
NPV	Negative predictive value = inverse precision	TN/(TN + FN)
MKD	Markedness	PPV + NPV– 1
**Precision-recall-type metrics**		
PRCN	Precision = PPV	TP/(TP + FP)
Recall	Recall	TP/AP
**Information-theoretic-type metrics**		
*Definitions*		
PrD1	Prior probability of a positive realization	TPn + FNn
PrD0	Prior probability of negative realization	1—PrD1
PrT1	Probability of a positive prediction	TPn + FPn
PrT0	Probability of a negative prediction	FNn + TNn
HD	Entropy of a realization = H(D)	−(PrD1×ln(PrD1)+PrD0×ln(PrD0))
HT	Entropy of a prediction = H(T)	−(PrT1×ln(PrT1)+PrT0×ln(PrT0))
HDT	Joint entropy = H(D, T)	−(TPn×ln(TPn)+FPn×ln(FPn)+FNn×ln(FNn)+TNn×ln(TNn))
IM	Expected mutual information = I_M_(D, T)	HD + HT–HDT
*Metrics*		
IMN	Normalized expected mutual information	IM/HD
MCEN	Modified confusion entropy	$\frac{2(FN+FP){log}_{2}((N-TN)(N-TP))}{3N+(FN+FP)}-\frac{4(FN{log}_{2}(FN)+FP{log}_{2}(FP))}{3N+(FN+FP)}$

#### Soft voting

A soft vote generally led to an ensemble with improved performance. For example, the soft vote ensemble was better than all but two of the base learners in terms of ROC-AUC (Fig 2C). However, improvement was also qualified by the metric on which performance was based. Soft voting improved Se over that of most of the base learners, but at the expense of Sp.

#### Model averaging

The *M*_*x*_ models also showed improved performance metrics over the base learners. As with soft voting, those improvements were not universal in all cases (Fig 2). The *M*_*x*_ models generally performed better in terms of Se than for Sp relative to the base learners (Fig 2A). The Se–Sp trade-off and a linear relationship between MKD and IFD were observed characteristics, also seen with the base learners. Seven of the 10 *M*_*x*_ models had a higher ROC-AUC than any of the base learners, and 34 of the 39 individual models had a lower ROC-AUC than the worst performing *M*_*x*_ model. From the information-theoretic perspective, the IMN score (higher is better) averaged over the *M*_*x*_ models was 0.123 in contrast to an average score of 0.095 over the base learners.

#### Stacking

The stacked models were superior to any other model analyzed in the current study based on multiple performance metrics (Fig 2). The average ROC-AUC computed over the three stacked models was 12.7% higher than the mean ROC-AUC over the individual logistic regression models, 7.8% higher than that of the soft vote model, and 6.9% higher than the mean ROC-AUC over the *M*_*x*_ models. In terms of PR-AUC, the performance gains due to stacking were found to be even greater, with 24%, 14% and 12% gains over the base learners, soft vote and *M*_*x*_ models, respectively. The trends seen in the plot of PR-AUC versus ROC-AUC (Fig 2C) were also seen qualitatively in the MKD vs IFD graph (Fig 2B), which is likely due to the high correlations of MKD and IFD with PR-AUC and ROC-AUC (S6 Fig). Stacked models with the lasso and elastic-net penalizations were nearly identical in ROC-AUC and PR-AUC performance measures (Fig 2C). There was a clear separation of the stacked regressions from all other models when evaluated on the composite IFD metric (which combines Se and Sp). However, looking at Se and Sp individually, the stacked regressions appeared to favor Sp over Se with the result that the Se of the stacked models were worse than that of the soft vote, several *M*_*x*_ models and some of the base learners.

Some finer points are worth mentioning. Although the MKD and IFD performance measures were linearly associated, the empirical results suggested a lower boundary to the relationship (Fig 2B), in that a small downward vertical shift in the regression line will place all points above it. With the information-theoretic metrics, IMN ranked the stacked models higher than any other model, unlike the MCEN metric (Fig 2D) where some other ensembles and a few base learners were ranked higher.

## Discussion

Model ensembling has been researched since the 1970s [1] yet has only recently been explored in some depth in disease epidemiology [4–6], including botanical epidemiology [7–9], although in the latter field there is some historical precedence for simple (non-statistical) combinations of usually no more than two forecasting models [24]. Ensembles typically combine models built using different algorithms, as this increases the diversity among the individual learners [1]. In this paper we showed that ensembling is still beneficial even when the individual learners are induced from the same algorithm (logistic regression) and the predicted probabilities are, in many cases, positively and highly correlated. Diversity in the predicted probabilities among the individual learners was therefore sufficient in this case study for ensembling to have an advantage and was generated by having the base learners trained on different subsets of the predictor feature space. Penalized stacking approaches, which addressed the correlations among the base learners, yielded the most benefit to ensembling in this situation.

Averaging simple models can lead to improved predictive performance [9], in general, but assumes that all models are independent (in their predictions) and equally plausible [25]. In practice, model independence (in terms of algorithmic construction, predictions, or both) is difficult to achieve. There was no single ‘optimal’ model among the 39 logistic regression models (base learners) given their predicted probabilities of epidemics, classification errors and performance metrics. The equal plausibility assumption may be reasonable for these base learners, but they clearly were related as shown by the positive correlations in their cv probabilities and Brier scores. As the correlations among base learner predictions increase, so does the overall prediction error in the ensemble which reduces the benefit gained from averaging (see Eq 5 in [26]). However, the fact that soft voting outperformed many of the base learners for the FHB data indicated that diversity was sufficient among the base learners to make this simple form of ensembling an effective strategy.

Ensembles seek to optimize predictive performance by capitalizing on reduced dependency and maximized diversity between models [27]; it is therefore best to understand how the base learners are related, particularly in how they are similarly wrong [11]. Our approach was to use hierarchical clustering on the dissimilarity matrix based on the Brier scores for 38 logistic regression models (eliminating one 1^st^ generation model because it was too often wrong). Models with similar Brier scores clustered together, indicating that they had the tendency to make the same errors. We postulated that little would have been gained from combining models within the same cluster, as model averaging performs best when done over dissimilar models [28]. In what amounts to essentially a subsampling and reweighting from the full set of logistic regression models [29], the dendrogram was split into groups under the assumption that models in the same group were too similar but models in different groups were less likely to be so. Choosing models across groups led to several base learner combinations that when averaged (weighted by the mean Brier scores of the models) resulted in better predictive performance than many (but not all) of the individual base learners, even though the combinations involved only four models each. These weighted averages of four models generally performed better than the soft voting (simple averaging) of all 38 base learners.

Larger performance gains were clearly realized with stacked regression [30] in contrast to both soft voting and weighted model averaging of small subsets of the base learners, despite the similarities among the 38 models. The meta-learner aspect of stacked regression finds the best weighted combination of the base learner predictions. We did initially use standard logistic regression as the meta-learner, only to obtain parameter estimates that were unstable as evidenced by unacceptably large standard errors. Penalized logistic regression (Eq 5) led to more stable meta-learner models [31] by shrinking the estimated parameters (ridge), setting some coefficient estimates to zero (lasso) or by mixing the ridge and lasso penalizations (elastic-net). There is the risk of overfitting both the base learners and the meta-learner [5]. However, this risk was reduced by having simple base learners, and by using nested cross-validation and penalization in training the meta-learner.

Predictive performance is only one goal in epidemiological forecasting; model interpretation is also important. With FHB, for example, understanding how disease develops or responds to environmental conditions is of fundamental epidemiological value, and although progress has been made in many aspects [32–34], much still has to be elucidated in a holistic framework [35]. Improved predictive performance due to ensembling is very encouraging, but it could be argued that meta-learning stills lags in being fully interpretable [36]. In the meantime, we may have to rely on interpretations of base learner models, but this may change given the progress being made in the interpretation of machine learning models [37,38]; and performance should not lose sight of its interpretive counterpart [39].

Our study was limited to one algorithmic form of base learner (logistic regression) heavily dependent on weather-derived predictors (albeit from different time windows) although variables for cultivar resistance to FHB and maize residue were included as baseline agronomic factors. Other FHB models have made use of other crop-related practices such as tillage and crop rotation [40] which reduce the amount of maize residue available as an inoculum source and hence as a risk factor of disease development. A data fusion approach [10] would develop base learners using specific types of data input sources, for example only weather data (as we have focused on) or built only with agronomically-relevant data, each model predicting the same target. These base models would then be ensembled. Learners can of course be induced by algorithms other than logistic regression [7,16]. Other approaches could include expanding the logistic regression models to include polynomial terms or generalizing to additive logistic regression in which the coefficients are no longer constants but functions themselves [15]. These were not pursued in the current study because the focus was on algorithms with low computational complexity and which were scalable, given the long-term goal of deploying FHB forecast models at a large spatiotemporal scale (multi-state). Moreover, rules other than Manhattan distance could be investigated in creating the dissimilarity matrix on which the family dendrogram of individual base learner models was predicated, as this affects the groupings upon which the *M*_*x*_ ensembles were drawn. Other ways of weighting the individual base learners in the *M*_*x*_ models could also be examined.

We limited our study to one type of response, a binary operational definition of FHB epidemics. Our models were also restricted to wheat production in the U.S. and even in that, do not cover the western States where FHB is much less common [41] and where field observations were not available. Other responses have been modeled in the FHB system, including grain contamination with the mycotoxins deoxynivalenol and zearalenone at harvest [40,42], indices of disease level or of mycotoxin concentration [34,43], ordinal representations of disease levels [44], and disease incidence directly [45]. These responses are clearly on different scales and represent different disease aspects (symptoms or toxin concentration, for example), and therefore ensembling across these models would be more challenging unless their disparate responses were somehow expressed in a common unit. Several empirical and theoretical approaches in botanical epidemiology for converting between disease response variables would be worth evaluating [46–48]. The disunity could be overcome by a common platform to foster collaboration among FHB (or other botanical epidemiological) working groups [5,6] and would further model development from different perspectives, which we believe will enhance ensembling efforts.

We close with some advisory words distilled for applied researchers, as ensemble methods and stacked generalization are not yet mainstream because of the associated computational complexity [38]. New software environments (e.g., the R sl3 package) will be helpful in automating or abstracting the fitting of ensembles. While the features of such tools are certainly appreciated, the onus is still on researchers to understand the characteristics of their data and representative models, and one must weigh whether an ensembling approach will help meet one’s objectives. The approach we have demonstrated is generalizable to any set of base learners (mechanistic, simulation, empirical, or mixture thereof) that exhibit highly correlated predictions on the same response variable. The generated set of base learner models are plausible descriptors of the response, but do not all make the same predictions (i.e., they make different errors) on the observations. If the base learners are highly correlated in terms of their predictions, one should recognize that relatively simplistic ensembling methods such as soft voting may not always lead to an ensemble that is better than the best-performing base learner. With many correlated models, a strategy of “overproduce and choose” may be pursued [49], which recognizes that it may be more parsimonious to only add models to an ensemble if they contribute meaningfully (however one defines it) to improving the ensemble. This is in essence what we did in conjunction with weighted model averaging, using a dendrogram approach to “prune” the ensemble on the full set of models to much smaller subsets without loss of predictive performance. Finally, one is free to use any algorithm in stacking the base learners, but linear models work well as the meta-learner [50]. If the base learners are highly correlated then it is judicious to use a penalized meta-learner in building the stacked ensemble model [31].

## Materials and methods

### Observational data

The data matrix consisted of 999 assessments of FHB in wheat, where the observations were made in research plots across multiple U.S. states. Plots received no fungicide treatment for disease control, and standard agronomic practices were followed for the area in which plots were located. FHB field severity (*S*), often called FHB index or disease index [51], was rated at wheat Feeke’s growth stage 11.1 [52] which is when the kernels are milky ripe. *S* is the mean percent of the wheat spike (head) surface area with symptoms. The research plots were in 17 U.S. states (AR, DE, IL, IN, KS, KY, MD, MI, MN, MO, ND, NE, NY, OH, PA, SD, WI) and had been established by the Integrated Management Coordinated Project of the U.S. Wheat & Barley Scab Initiative. In general, there is only a narrow window for assessing disease, about 18 to 21 days after wheat anthesis; by 7 to 14 days later, the plant senesces, and disease symptoms are no longer clearly discernable from natural senescence. Plot data were available in 32 years from 1982 to 2015; not all 17 states were represented in each of those years. Besides *S*, other plot-level data recorded were wheat type (i.e., wheat market class) [spring (265 observations) or winter (734 observations)]; cultivar resistance class [representing different FHB susceptibility levels: very susceptible (135 observations), susceptible (412 observations), moderately susceptible (213 observations), and moderately resistant (239 observations)]; anthesis date (visible flowering, anthers extruded on at least 50% of the spikes in a plot); and the presence (348 observations) or absence (647 observations) of maize residue within plots or immediately next to the plots (4 observations missing such data). Maize residue is relevant because maize is a host on which the pathogen *Fusarium graminearum* can survive and grow between and within seasons [53]. Inoculum (spores) of the pathogen is produced on both wheat and maize.

### The response variable

As with all our past work, the continuous variable *S* (on a 0 to 100 percentage scale) was dichotomized to a binary classification variable *y*, where

$$y_{i}=\left\{ \begin{array}{c} 0\;if\;S_{i}<10\\ 1\;if\;S_{i}\geq 10 \end{array}\right.$$ (1)

for the *i*^th^ observation. That is, *y*_*i*_ were realizations of the random variable *Y*_*i*_ representing whether the *i*^th^ observation was of high or (relatively) low disease severity, operationally viewed as a major or non-major FHB epidemic (hereafter referred to as epidemic and non-epidemic for convenience). This operational definition based on *S* translates to economically important thresholds for mycotoxin contamination [47] and yield reduction [54], and serves as the basis for risk predictions in the U.S. National FHB Prediction Tool.

Models (described below) attempt to predict the expected value of *Y*_*i*_ (i.e., *E*(*Y*_*i*_)) which equaled the probability *p*_*i*_ that the *i*^th^ observation was of an epidemic. Our model framework was standard logistic regression, so that

$$g[E(Y_{i})]=\mu +\beta_{1}X_{1i}+\cdots +\beta_{h}X_{hi}$$ (2)

where *μ* was the overall intercept, the *β*_*j*_ were regression coefficients associated with each of the *h* predictors in the model, and *g*(.) was the logit link function, log(*p*_*i*_/(1−*p*_*i*_)), so that Eq 2 was linear with respect to the predictors on the logit scale.

### Scalar predictors

The logistic regression models we had published to date [7,15,16] had varied in the categorical (agronomic) predictors they included. All typically included a predictor for the level of susceptibility to FHB but may not have included predictors for wheat type (spring or winter wheat) or for the presence (absence) of maize residue (a potential source of the pathogen). The following categorical (factor) predictors were used in all of the logistic regression models in the current article: (i) *rs*, with four levels representing cultivar susceptibility to FHB (very susceptible, susceptible, moderately susceptible, moderately resistant, where the definition of susceptibility was based on locally-adapted standard susceptible and resistant checks, i.e., reference cultivars); and (ii) *wc*, a three-level variable reflecting wheat agronomic practices with respect to maize residue (spring wheat, winter wheat with maize residue, winter wheat in the absence of maize residue).

### Weather-based predictors

**Fig 3:**
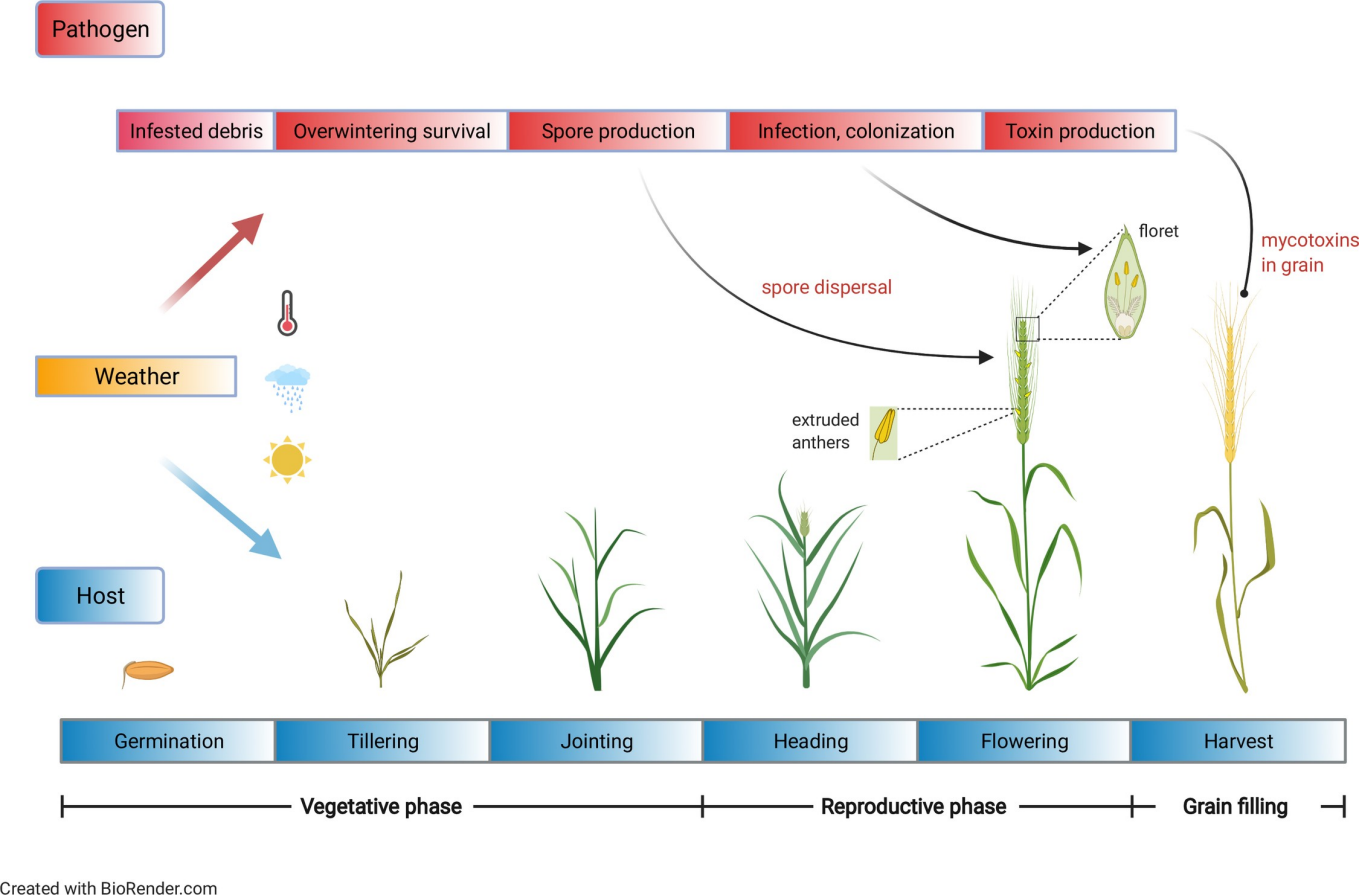
Schematic of wheat growth phases and key stages in the life cycle of *Fusarium graminearum* which causes Fusarium head blight. Wheat growth and development as well as pathogen survival, reproduction, dispersal, and infection are all affected by weather. Spores must land on the wheat spike (head) sometime between flowering and early grain fill, which is the period of greatest host susceptibility (but is also of limited duration) for infection. Successful infection and colonization of the spike is associated with mycotoxin accumulation in the grain.

Our 1^st^ and 2^nd^ generation FHB epidemic classifiers [7,13,15] were driven by variables summarizing temperature and moisture (relative humidity or vapor pressure deficit) in windows no more than 15 days either side of anthesis, for a few key reasons. Modeling had to be cognizant of the fact that fungicide applications must be made at anthesis or no more than five days after anthesis to control the disease effectively [55], as infections of the spike by the fungus occur primarily during flowering [56]. As FHB epidemics are weather-driven, it was logical to summarize meteorological conditions close to anthesis (Fig 3). Among other things, many of the *F*. *graminearum* spores infecting wheat at anthesis may be produced in a relatively short period before anthesis [32]. Spore production and dispersal, and infection of spikes are all functions of environmental conditions, especially moisture and temperature in certain ranges [57,58]. More extensive statistical queries showed that the strongest associations between weather summaries and FHB occurred in short windows surrounding anthesis [59], thereby reinforcing earlier intuitions.

### Third generation models

Functional regressions on weather series from 120 days pre-anthesis to 30 days post-anthesis [14,16] further showed that signals associated with FHB epidemics could be found as early as 40 to 60 days pre-anthesis; yet at the same time confirming that the strongest signals were at or around flowering. After examining the functional regression results, 92 novel weather-based candidate predictor variables were postulated, summarizing hourly or daily conditions during periods statistically associated with epidemics. This latter set of functional-regression-inspired predictor variables had not been used in any of the 1^st^ or 2^nd^ generation models [7,13,15], and furthermore were not restricted to the 15-day windows on either side of anthesis as had been the case in the earlier models.

Weather-derived variables tend to be highly correlated [15], partly due to them being defined over similar time periods relative to anthesis and also due to inter-relationships (e.g., both relative humidity and vapor pressure deficit are calculated from air temperature and the dewpoint). High correlations among predictor variables can be problematic for standard logistic regression (e.g., high variance associated with estimated parameters).

For the above reasons, the novel proposed set of 92 variables was screened before being considered for logistic regression models. First, predictors were checked for low to negligible separation of epidemics and non-epidemics (via distributional plots of the predictor variable by epidemic class), which culled eight variables from further consideration. The remaining 84 variables did not exhibit extreme collinearity [60]. The 84 variables were then grouped according to whether they represented conditions in the pre-anthesis period (49 variables), the post-anthesis period (18 variables), or any period relative to anthesis (17 variables), which reflected efforts to model epidemics as a function of the pre-anthesis window only, the post-anthesis window only, or with windows crossing anthesis (i.e., pre-to-post anthesis) [15]. Post and pre-to-post anthesis models may provide predictions too late for fungicide application decisions to be made, but are useful for other FHB management strategies related to grain harvesting, marketing, and the supply chain [22,61].

The three groups of predictor variables were independently screened by two machine-learning algorithms: lasso regression and the relative influence measure from a boosted regression tree [62] fit to the *y_i_*. Lasso regression performs variable selection by setting the coefficients of ‘unimportant’ variables to zero, a form of regularization. The relative influence measure from boosted regression estimates the importance of a variable to prediction. The lasso λ parameter was tuned via 10-fold cross-validation (described later) using binomial deviance as the loss measure. Boosted regression trees were tuned by a grid search over tree depth (2 or 3), the number of trees (1,000 to 3,500), and the shrinkage parameter (0.005 to 0.015), while the minimum terminal node size and bag fraction were held at 10 and 0.75, respectively. The eighteen (out of 49) pre-anthesis variables selected by the lasso were input into the boosted regressions. As lasso aggressively culled the post-anthesis and pre-to-post anthesis variables to three each, the two latter sets of predictor variables (18 and 17, respectively) were input directly into boosted regression without the lasso pre-selection step as was done with the pre-anthesis variables. The variables were sorted by their relative influence scores returned by the tuned boosted regressions done independently on each of the three sets (i.e., 18 pre-anthesis variables, 18 post-anthesis variables and 17 pre-to-post anthesis variables). This latter total set of 53 variables represented a greater diversity in terms of weather conditions, summary measure and windows relative to anthesis (S1 Table) than was present in the models up to 2014.

#### Feature set partitioning

**Fig 4:**
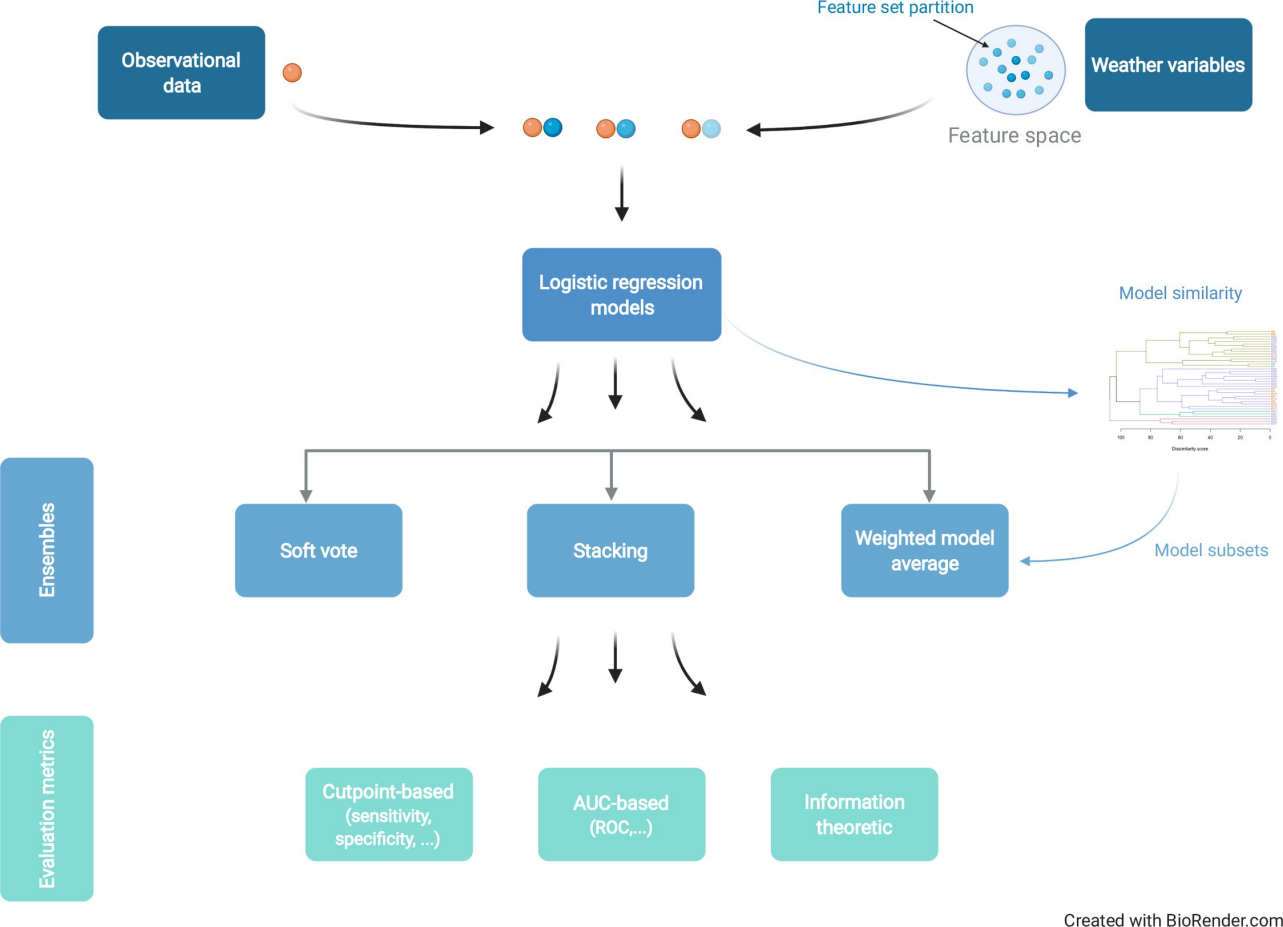
Schematic of the analytical steps. The observational data (orange sphere) are linked to weather-based predictors, the full set of the latter (feature space) having been partitioned into smaller subsets of one to three variables each (blue spheres). The datasets (orange-blue sphere combinations) are used to train logistic regression models (base learners). The base learners are then ensembled using one of three methods. Whereas the soft vote and stacking methods ensemble all base learners, the weighted model average uses a smaller subset of the base learners chosen to capitalize on diversity within the subset. All models are then evaluated using metrics which fall into three broad categories. Cut-point based metrics are calculated after conversion of the fitted probabilities to a classification. Area under the curve (AUC) metrics summarize performance over all possible cut-points and do not rely on any single such point. Information-theoretic metrics are based on concepts such as entropy.

Within the pre-, post- and pre-to-post anthesis groups, the relative influence-sorted variables were split into subsets of two or three variables each, starting with the variable with the highest relative influence and working down the list, conditional on (i) the subset not having two variables summarizing the same type of weather (e.g., two temperature variables), and (ii) any two variables within a subset not having a pairwise Pearson correlation above 0.9. Subsets of one variable were not considered here, and larger subsets were less likely to meet the two conditions stipulated above. The two lasso post-anthesis and pre-to-post anthesis subsets of three variables each were also retained. The 24 subsets so created via the machine-learning screenings were used as the input weather-based variables for a new generation of logistic regression models; these are considered along with the previously developed models for forecasting FHB (Fig 4).

### Model genealogy

For the current article we analyzed 39 logistic regression models, which consisted of: three first-generation models [13]; 12 second-generation models [7,15], and 24 third-generation models, four of which were described previously [16] and the rest described in S2 Table. These 39 models made use of 77 different weather-based predictor variables, in which six, eight, and 25 models had one, two, and three weather-based predictors, respectively.

### Model fitting and evaluation

There were 273 observations of FHB epidemics (as defined by Eq 1) out of 999 total observations. Ten-fold cross-validation (cv) was used to obtain estimates of model performance. For the cv procedure, the full dataset was divided randomly into 10 (approximately) equal-sized samples (folds). Holding out each fold in turn, models were trained on the data in the remaining nine folds, and the fitted models then used to obtain the predicted probabilities on the held-out fold. Fitted probabilities were obtained for each of the observations by iterating through this algorithm with each fold serving as a test set.

The cv probabilities were converted to predicted classifications using the respective predicted-probability cut-point that maximized the Youden Index (YI; sensitivity + specificity – 1) for each model *m* (*c*_*m*_) to arrive at classifications [63]. Cut-points were estimated independently for each model, given the cv probabilities (hence the *m* subscript on *c*).

#### Performance metrics

A plethora of metrics exists for evaluating the performance of binary classification models, with no clear consensus [64]. We concentrated on a set of performance metrics that included some traditional ones used in machine learning, as well as a few others that were recently proposed or discussed (Table 1). Here cut-point-based means that the confusion matrix (and metrics summarizing it) depend on the cut-point used for converting the estimated probabilities to a class membership. Powers [23] presented informedness (IFD) and markedness (MKD). From our viewpoint, they were attractive measures because taken together they summarize the confusion matrix in both the column-wise (IFD) and row-wise (MKD) directions. In binary classification (as done here), IFD is the same (algebraically) as both YI (= *J* statistic) and the axiomatically derived *K* measure [65]. Two information-theoretic metrics we included were the normalized expected mutual information (IMN), which is equivalent to McFadden’s R^2^ [66]; and the recently proposed modified confusion entropy (MCEN; [67]). The priors for IMN were taken as the proportion of epidemics and non-epidemics in the full dataset. Higher values of IMN (because of the normalization) indicate better classification performance, whereas lower values of the entropy-based MCEN are indicative of better classification. The final two metrics, which are from the family of ranking measures [64], were the area under the receiver operating characteristic curve (ROC-AUC), and the area under the precision-recall curve (PR-AUC), which are of course independent of cut-point.

#### Brier scores

The Brier score, a proper scoring rule [68], was used to summarize how close a model’s cv probabilities were to the real (observed) class memberships. Upon fitting Eq 2, the cv probability for observation *i* in a held-out fold was given by

$${\hat{p}}_{i}=\frac{e^{\mu }+\beta_{1}X_{1i}+\cdots +\beta_{h}X_{hi}}{1+e^{\mu +\beta_{1}X_{1i}+\cdots +\beta_{h}X_{hi}}}$$ (3)

The Brier score for observation *i* for a single logistic model was estimated as $B_{i}={\left({\hat{p}}_{i}-y_{i}\right)}^{2}$, where *y*_*i*_ = 0 for a non-epidemic observation and *y*_*i*_ = 1 for an epidemic observation. Scores were calculated for the cv probabilities returned by each logistic regression model *m*, so that we have *B*_*m*,*i*_, *m* = 1, …, 39, *i* = 1, …, 999. The mean Brier score for each model *m*, ${\bar{B}}_{m}=\frac{1}{999}\sum_{i=1}^{999}B_{m,i}$, was also calculated. The *B*_*m*,*i*_ and ${\bar{B}}_{m}$ scores were used for understanding the similarities and variability between and across models.

### Model ensembles

Three different methods of ensembling the individual logistic regression models were investigated: soft voting, weighted model averaging and stacking (Fig 4). Models are referenced by their index (e.g., *m* = 5) or their label (e.g., M5). Model M3 was not included in ensembling as it was an obvious outlier (poor model fit; see Results) compared with the other 38 models. M3 was a first-generation model originally developed with only 50 observations [13]. Dropping M3 followed the principle of eliminating poor performers in a model genealogy [12].

#### Soft voting

For each observation *i*, the cv probabilities from the individual 38 logistic regression models were simply averaged to obtain the soft vote probability (${\hat{p}}_{(sv),i}=\frac{1}{38}\sum_{m=1}^{38}{\hat{p}}_{m,i}$). This represented the reference approach to the ensembling methods investigated in the current article, as no consideration was given to the differences in predictive ability (across all observations) among the individual models [26]. The soft vote model classification was then based on the cut-point (*c*_*sv*_) for which YI was maximal given the ${\hat{p}}_{(sv),i}$.

#### Model averaging

A 38 × 38 dissimilarity matrix of the models was produced from the *B*_*m*,*i*_ scores using the Manhattan distance metric, which is the absolute distance between two vectors (L^1^ norm). The dissimilarity matrix was input into a hierarchical clustering algorithm to give a ‘family tree’ of the models [27]. The resulting dendrogram was, upon inspection, cut into four groups consisting of 15, 17, 3 and 3 models, respectively, reflecting their similarities in terms of their Brier scores.

Let *G*_*lj*_ represent the *l*^th^ model (*l* = 1, …, *g*_*bj*_) in the *j*^th^ group (*j* = 1, …, 4) from the cluster analysis, where *G*_•*j*_ refers to the model group. For example, for group 1, *g*_*b*1_ = 15 models in the group and *G*_51_ is the 5^th^ model in *G*_•1_. In the next stage, one logistic regression model was taken from each *G*_•*j*_ to give a set *M*_*x*_ consisting of four individual logistic regression models. For the model ensembles, it was assumed that the logistic regression models within a *G*_•*j*_ were too alike in terms of cv probabilities for the same observations (the basis for the dendrogram), but that models from *G*_•*j*_ and *G*_•*k*_ (*j* ≠ *k*) were less likely to be as closely related. Given four *G*_•*j*_ of size 15, 17, 3 and 3, there were 2,295 possible unique *M*_*x*_ sets; we chose 10 of them at random, (i.e., *x* = 1, …, 10).

For each *M*_*x*_, the cv probabilities of epidemics of the four constituent base learner models were combined using a weighted average, where the weights *w*_*m′*_(*M*_*x*_) were estimated from the mean Brier scores, ${\bar{B}}_{m^{\prime }}$, of the four individual models (*m*′ = 1, …4; *m*′ being a subset of *m*):

$$w_{m^{\prime }}(M_{x})=\frac{exp(-0.5\;{\bar{B}}_{m^{\prime }})}{\sum_{m^{\prime }=1}^{4}{exp(-0.5\;\bar{B}}_{m^{\prime }})}$$ (4)

The weighted combined cv probability of an epidemic was taken as the model-averaged probability returned for each *M*_*x*_ for any given observation *i*. After obtaining the estimated probabilities for a *M*_*x*_ model, the cut-point which maximized YI ($c_{M_{x}}$) was used to generate the confusion matrix from which the associated performance metrics were then calculated.

#### Stacking

The base learner models were the 38 simple logistic regression models. The stacking algorithm we used, in general terms, was as follows. The cv probabilities of the 38 base learners were collected into a *I* × *L* matrix. In the present context *I* = 999 (the number of observations) and *L* = 38. The *I* × *L* matrix was augmented with a column representing the responses (0s and 1s representing non-epidemics and epidemics, respectively; i.e, *y*_*i*_). In stacking terminology, this augmented matrix is called the Level 1 data. The Level 1 data were then used to train a meta-learning algorithm, in which the *y*_*i*_ were modeled as a function of the cv probabilities of epidemics returned by the 38 base learners:

$$g[E(Y_{i})]=\theta +\delta_{1}{\hat{p}}_{1i}+\cdots +\delta_{38}{\hat{p}}_{38i}$$ (5)

where *g*(.) was the logit link function (as in Eq 2), *θ* was the overall intercept, and the *δ* were the coefficients for the predicted cv probabilities ($\hat{p}$) for the 38 base learners. We used penalized logistic regression with ridge, lasso or elastic-net penalties as the meta-learner, thus staying within a logistic regression framework in estimating the *δ* coefficients. Penalization was used because of the high correlations among the base learner cv probabilities of epidemics, to prevent overfitting and to improve the overall generalization accuracy [31]. The elastic-net alpha tuning parameter was set to 0.5, so that correlated Level 1 predictors would be selected or removed together. For all three forms of penalization, the lambda parameter controlling the amount of penalization was tuned via *k*-fold cross-validation [69]. The trained meta-learners were then used for prediction, where the inputs to the meta-learner were the cv probabilities of epidemics for each observation returned by the base learners weighted by the *δ* coefficients estimated for each of the three penalization methods.

In more detail, a nested cv procedure was used to avoid training and evaluating the meta-learners on the same data [38], which can lead to so-called data leakage and potentially overoptimistic estimates of model test performance [26,70]. Let *X* represent the data matrix consisting of the response vector plus columns for the categorical and weather-based predictors used by each of the base learners. In the algorithm that follows, we follow the terminology proposed by Kuhn and Johnson [71], and use the terms “analysis” and “assessment” to describe the resampling of data into subsets used for model development and tuning (analysis), and for measuring model performance (assessment). These terms are analogous to traditional training and testing partitioning but occur within a resampling framework such as cross-validation. The pseudocode is as follows:

1. Split *X* into 10 cv folds (*k*_*o*_; *k*_*o*_ = 1, …, 10), where each *k*_*o*_ consisted of an analysis partition (90% of the data, about 899 observations) and an assessment partition (the remaining 10% of the data, about 100 observations). This constituted the outer resample (hence the *o* subscript on *k*). For each *k*_*o*_:
  - Train the base learners on the analysis partition.
  - Use the trained base learners to obtain cv probabilities of epidemics on the assessment partition. These outer resample probabilities will be used as the input variables to the trained meta-learner (Step 3.b).
2. Within each *k*_*o*_:
  - Further split the analysis partition into five folds (*k*_*i(o)*_; *i* = 1, …, 5). This was the inner (nested) resample (hence the *i* subscript on *k*). Each *k*_*i(o)*_ fold was likewise made up of an analysis (about 719 observations) and an assessment partition (about 180 observations), the total number of observations being equivalent to the number of observations in the respective *k*_*o*_ analysis fold. The inner analysis and assessment folds were used to train the meta-learner. For each *k*_*i(o)*_:
    - Fit each of the 38 base learners on the analysis partition data.
    - Use the fitted models to obtain the predicted cv probabilities of epidemics on the respective inner assessment data.
  - Assemble the cv probabilities from the five *k*_*i(o)*_. These predicted probabilities (38 columns, one for each base learner) on the inner assessment partitions plus the associated response vector (i.e, the *y*_*i*_ observations) constituted the Level 1 data. Each Level 1 matrix was therefore ~899 rows (depending on the number of observations in *k*_*o*_ from Step 1) and 39 columns.
3. Because of the nesting, 10 versions of the meta-learner were trained. That is, for each *k*_*o*_:
  - Train the meta-learner (i.e., fit Eq 5). Determine the value of the tuning parameter via 10-fold cross-validation on the respective Level 1 data matrix.
  - Use the trained meta-learner (fitted Eq 5) with the outer resample cv probabilities (Step 1.b) as the input to predict the probability of an epidemic for an observation.
4. Collect the predicted probabilities from Step 3.b (about 100 per *k*_*o*_). These are the predicted cv probabilities of an epidemic returned by the meta-learner.

The cut-point which maximized YI given the probabilities returned in Step 4 was used to generate the confusion matrix from which performance metrics were then estimated for the penalized meta-learners, as done with the previous analyses (above).

### Software and code

All analyses were done with R version 3.5.3 (2019-03-11). The λ penalization parameter for lasso, ridge and elastic-net was tuned via 10-fold cross-validation using the cv.glmnet function in the glmnet package (version 2.0–16) with binomial deviance as the loss measure. Training and tuning of boosted regression trees were carried out using the caret package (version 6.0–82) as a wrapper to the gbm function of the gbm package (version 2.1.5). The cross-validation procedure was programmed using the train function in the caret package as a wrapper to the generalized linear model (glm) function for fitting Eq 2. Hierarchical clustering was done with the hclust function using the complete agglomeration method. The data and code for reproducing the analyses are available via the Dryad Digital Repository: https://doi.org/10.5061/dryad.fn2z34trv [72].

## Supporting information

S1 Table Weather-based predictors used in logistic regression models for the occurrence of Fusarium head blight epidemics. D, dewpoint (°C); P, barometric pressure (hPa); VPD, vapor pressure deficit (kPa); RH, relative humidity (%); T, air temperature (°C); TDD, temperature-dewpoint depression (°C); sd, standard deviation.
(DOCX)

S2 Table Descriptions of logistic regression models used for predicting epidemics of Fusarium head blight. pre, weather variables summarize conditions from pre-anthesis to anthesis; post, weather variables summarize conditions from anthesis to post- anthesis; pre-to-post, weather variables summarize conditions starting pre-flowering and ending post-flowering. ^a^ 1^st^-generation models were described in De Wolf et. al. (2003), 2^nd^-generation models in Shah et. al. (2013, 2014). Four 3^rd^-generation models (M16-M19) were described in Shah et. al. (2019), with the remaining 3^rd^-generation models being described in the current study. The originally published version of model M3 did include a precipitation variable. However, the precipitation variable was not included here, and none of the other models in the Table use precipitation-derived variables. ^b^ See S1 Table.
(DOCX)

S1 Fig Correlation matrix of the Pearson correlation between cross-validated fitted probabilities of an epidemic for the 39 logistic regression models. The label colors indicate what generation the model belongs to: green, 1^st^ generation; orange, 2^nd^ generation; purple, 3^rd^ generation.
(TIF)

S2 Fig Empirical distributions of the predicted probability of epidemics (based on cross-validation) returned by the 39 logistic regression models for observations 251 to 275. Epi, observation was an epidemic; Nonepi, observation was a non-epidemic. There is a separate panel for each observation. Cultivar resistance levels to Fusarium head blight were VS, very susceptible; S, susceptible; MS, moderately susceptible; MR, moderately resistant. The chosen observations were an arbitrary sample of 25 from the 999 to demonstrate the diversity of distributional results for epidemic predictions. The vertical dashed line in each panel represents the proportion of observations that were FHB epidemics in the data (0.27).
(TIF)

S3 Fig Classification of observations 251 to 275 by 39 logistic regression models. Epi, observation was an epidemic; Nonepi, observation was a non-epidemic. There is a separate panel for each observation. The data points in each panel represent the epidemic classifications by each of the logistic regression models, based on dichotomizing the predicted probability (from cross validation) of an epidemic. For each model the cut-point for classification was that for which the Youden Index was maximal. Correct, observation was correctly classified by the model; FP, observation’s classification was a false positive; FN, observation’s classification was a false negative. Cultivar resistance classes to Fusarium head blight were VS, very susceptible; S, susceptible; MS, moderately susceptible; MR, moderately resistant. The observations are the same arbitrary sample as in S2 Fig to show the diversity of results.
(TIF)

S4 Fig Correlation matrix of the Pearson correlation between Brier scores for the 39 logistic regression models. Brier scores were calculated for the predictions of epidemics based on cross-validated fitted probabilities returned by the models for each observation. For each pair of models, the Pearson correlation was calculated between the Brier scores for the 999 observations. The label colors indicate what generation the model belongs to: green, 1^st^ generation; orange, 2^nd^ generation; purple, 3^rd^ generation.
(TIF)

S5 Fig The mean Brier score for the logistic regression models. For each of the 39 models, the mean Brier scores were calculated over all observations in the dataset, based on the predicted probabilities of an epidemic (using the cross-validated fitted probabilities). The mean Brier scores are sorted. The dashed line is at the overall mean of 0.171. Mean Brier scores decrease with improving cross-validated fit to the data. The label and point colors indicate what generation the model belongs to: green, 1^st^ generation; orange, 2^nd^ generation; purple, 3^rd^ generation.
(TIF)

S6 Fig Relationships and Pearson correlations between performance metrics using the cut-point which maximized the Youden Index. Each graphics panel displays the results for 39 logistic regression models (S2 Table). Metric definitions are in Table 1.
(TIF)
