# Supplementary material for: Accuracy in the prediction of disease epidemics when ensembling simple but highly correlated models
Source: PLoS Comput Biol. 2021 Mar 15;17(3):e1008831. doi: 10.1371/journal.pcbi.1008831 (PMC7993824; doi:10.1371/journal.pcbi.1008831)
Supplement: S1 Table — D, dewpoint (°C); P, barometric pressure (hPa); VPD, vapor pressure deficit (kPa); RH, relative humidity (%); T, air temperature (°C); TDD, temperature-dewpoint depression (°C); sd, standard deviation. (DOCX) [file pcbi.1008831.s001.docx]

| **Weather variable** | **Description** |
| --- | --- |
| D.A.1 | Mean D from 20 days pre-anthesis to anthesis |
| D.A.2 | Mean D from 20 days pre-anthesis to 10 days pre-anthesis |
| D.MINMAXDIFF.3 | Mean of daily max - min D from 20 days pre-anthesis to 10 days post-anthesis |
| D.SD.3 | Mean daily sd(D) from 40 days pre-anthesis to 10 days post-anthesis |
| INT3 | T15307 × TRH9010 interaction (product term) |
| P.A.1 | Mean P from 40 days pre-anthesis to 20 days pre-anthesis |
| P.A.2 | Mean P from 45 days pre-anthesis to 35 days pre-anthesis |
| P.A.3 | Mean P from 60 days pre-anthesis to 50 days pre-anthesis |
| P.A.5 | Mean P from 100 days pre-anthesis to 60 days pre-anthesis |
| P.MINMAXDIFF.1 | Mean of daily max - min P from 60 days pre-anthesis to 50 days pre-anthesis |
| RH.A.1 | Mean RH from 20 days pre-anthesis to anthesis |
| RH.A.3 | Mean RH from 10 days pre-anthesis to 10 days post-anthesis |
| RH.A.POST10.12H | Mean overnight RH from anthesis to 10 days post-anthesis |
| RH.A.POST5.12H | Mean overnight RH from anthesis to 5 days post-anthesis |
| RH.A.POST7.12H | Mean overnight RH from anthesis to 7 days post-anthesis |
| RH.A.PRE10.24H | Mean RH from 10 days pre-anthesis to anthesis |
| RH.A.PRE14.24H | Mean RH from 14 days pre-anthesis to anthesis |
| RH.G70.CD.2 | No. of days daily mean RH > 70% from anthesis to 10 days post-anthesis |
| RH.G70.CD.3 | No. of days daily mean RH > 70% from 15 days pre-anthesis to 10 days post-anthesis |
| RH.G80.CHD.2 | No. of h RH ≥ 80% from anthesis to 10 days post-anthesis |
| RH.G80.CHD.3 | No. of h RH ≥ 80% from 20 days pre-anthesis to 10 days post-anthesis |
| RH.G80.PRE14.12H | No. of overnight h RH ≥ 80% from 14 days pre-anthesis to anthesis |
| RH.G90.CHD.1 | No. of h RH ≥ 90% from 20 days pre-anthesis to anthesis |
| RH.G90.CHD.2 | No. of h RH ≥ 90% from anthesis to 10 days post-anthesis |
| RH.G90.CHD.3 | No. of h RH ≥ 90% from 50 days pre-anthesis to 10 days post-anthesis |
| RH.G90.PRE10.12H | No. of h overnight RH ≥ 90% from 10 days pre-anthesis to anthesis |
| RH.MINMAXDIFF.3 | Mean of daily max - min RH from 30 days pre-anthesis to 10 days post-anthesis |
| RH7 | Mean RH from 7 days pre-anthesis to anthesis |
| sq.T.A.PRE7.24H | The square of T.A.PRE7.24H |
| T.A.1 | Mean T from 25 days pre-anthesis to 15 days pre-anthesis |
| T.A.2 | Mean T from 20 days pre-anthesis to 10 days pre-anthesis |
| T.A.3 | Mean T from 40 days pre-anthesis to 30 days pre-anthesis |
| T.A.4 | Mean T from anthesis to 10 days post-anthesis |
| T.A.5 | Mean T from anthesis to 20 days post-anthesis |
| T.A.POST5.24H | Mean T from anthesis to 5 days post-anthesis |
| T.A.POST7.24H | Mean T from anthesis to 7 days post-anthesis |
| T.A.PRE15.24H | Mean T from 15 days pre-anthesis to anthesis |
| T.A.PRE7.24H | Mean T from 7 days pre-anthesis to anthesis |
| T.G30.CHD.3 | No. of h T > 30°C from 10 days post-anthesis to 20 days post-anthesis |
| T.L9.PRE15.24H | No. of h T < 9°C from 15 days pre-anthesis to anthesis |
| T.L9.PRE7.24H | No. of h T < 9°C from 7 days pre-anthesis to anthesis |
| T.MINMAXDIFF.2 | Mean of daily max - min T from 20 days pre-anthesis to 10 days post-anthesis |
| T.SD.1 | Mean daily sd(T) from 20 days pre-anthesis to anthesis |
| T.SD.2 | Mean daily sd(T) from anthesis to 10 days post-anthesis |
| T.SD.3 | Mean daily sd(T) from 20 days pre-anthesis to 10 days post-anthesis |
| T15307 | No. of h 15°C ≤ T ≤ 30°C from 7 days pre-anthesis to anthesis |
| TDD.A.2 | Mean TDD from 30 days pre-anthesis to anthesis |
| TDD.A.4 | Mean TDD from anthesis to 20 days post-anthesis |
| TDD.A.5 | Mean TDD from 10 days pre-anthesis to 5 days post-anthesis |
| TDD.A.6 | Mean TDD from 10 days pre-anthesis to 10 days post-anthesis |
| TDD.SD.3 | Mean daily sd(TDD) from anthesis to 10 days post-anthesis |
| TDD.SD.4 | Mean daily sd(TDD) from 20 days pre-anthesis to 10 days post-anthesis |
| TRH.15T30nRHG80.CHD.1 | No. of h 15°C ≤ T ≤ 30°C & RH ≥ 80%, 30 days pre-anthesis to anthesis |
| TRH.15T30nRHG80.CHD.2 | No. of h 15°C ≤ T ≤ 30°C & RH ≥ 80%, 60 to 40 days pre-anthesis |
| TRH.15T30nRHG80.CHD.3 | No. of h 15°C ≤ T ≤ 30°C & RH ≥ 80%, anthesis to 10 days post-anthesis |
| TRH.15T30nRHG80.POST10.12H | No. of overnight h 15°C ≤ T ≤ 30°C & RH ≥ 80%, anthesis to 10 days post-anthesis |
| TRH.15T30nRHG80.POST5.12H | No. of overnight h 15°C ≤ T ≤ 30°C & RH ≥ 80%, anthesis to 5 days post-anthesis |
| TRH.15T30nRHG80.POST7.12H | No. of overnight h 15°C ≤ T ≤ 30°C & RH ≥ 80%, anthesis to 7 days post-anthesis |
| TRH.15T30nRHG80.PRE15.24H | No. of h 15°C ≤ T ≤ 30°C & RH ≥ 80%, 15 days pre-anthesis to anthesis |
| TRH.15T30nRHG90.CHD.1 | No. of h 15°C ≤ T ≤ 30°C & RH ≥ 90%, 30 days pre-anthesis to anthesis |
| TRH.15T30nRHG90.CHD.3 | No. of h 15°C ≤ T ≤ 30°C & RH ≥ 90%, anthesis to 10 days post-anthesis |
| TRH.5T30nRHG75.CHD.3 | No. of h 5°C ≤ T ≤ 30°C & RH ≥ 75%, anthesis to 10 days post-anthesis |
| TRH.9T30nRHG90.CHD.2 | No. of h 9°C ≤ T ≤ 30°C & RH ≥ 90%, 50 days pre-anthesis to anthesis |
| TRH.9T30nRHG90.PRE15.24H | No. of h 9°C ≤ T ≤ 30°C & RH ≥ 90%, 15 days pre-anthesis to anthesis |
| TRH9010 | No. of h 15°C ≤ T ≤ 30°C & RH ≥ 90%, anthesis to 10 days post-anthesis |
| VPD.A.1 | Mean VPD from 10 days pre-anthesis to anthesis |
| VPD.A.3 | Mean VPD from anthesis to 15 days post-anthesis |
| VPD.A.4 | Mean VPD from anthesis to 20 days post-anthesis |
| VPD.A.5 | Mean VPD from 10 days pre-anthesis to 10 days post-anthesis |
| VPD.L11.CHD.2 | No. of h VPD ≤ 1.1 kPa, 40 days pre-anthesis to anthesis |
| VPD.L45.PRE7.12H | No. of overnight h VPD ≤ 0.45 kPa, 7 days pre-anthesis to anthesis |
| VPD.L6.CD.1 | No. of days daily mean VPD < 0.6 kPa, 20 days pre-anthesis to anthesis |
| VPD.L6.CD.3 | No. of days daily mean VPD < 0.6 kPa, anthesis to 20 days post-anthesis |
| VPD.L635.CHD.1 | No. of h VPD ≤ 0.635 kPa, 15 days pre-anthesis to 5 days pre-anthesis |
| VPD.L635.CHD.2 | No. of h VPD ≤ 0.635 kPa, 40 days pre-anthesis to 5 days pre-anthesis |
| VPD.SD.3 | Mean daily sd(VPD) from anthesis to 10 days post-anthesis |
| VPD.SD.4 | Mean daily sd(VPD) from 10 days pre-anthesis to 10 days post-anthesis |
