# Supplementary material for: Accuracy in the prediction of disease epidemics when ensembling simple but highly correlated models
Source: PLoS Comput Biol. 2021 Mar 15;17(3):e1008831. doi: 10.1371/journal.pcbi.1008831 (PMC7993824; doi:10.1371/journal.pcbi.1008831)
Supplement: S2 Table — a 1st-generation models were described in De Wolf et. al. (2003), 2nd-generation models in Shah et. al. (2013, 2014). Four 3rd-generation models (M16-M19) were described in Shah et. al. (2019), with the remaining 3rd-generation models being described in the current study. The originally published version of model M3 did include a precipitation variable. However, the precipitation variable was not included here, and none of the other models in the Table use precipitation-derived variables. b See S1 Table. (DOCX) [file pcbi.1008831.s002.docx]

| **Model** | **Generation**^a^ | **Weather-based predictors^b^** | **Period** |
| --- | --- | --- | --- |
| M1 | 1^st^ | TRH9010 | post |
| M2 | 1^st^ | INT3 | pre-to-post |
| M3 | 1^st^ | T15307 | pre |
| M4 | 2^nd^ | RH7 | pre |
| M5 | 2^nd^ | RH.A.PRE10.24H | pre |
| M6 | 2^nd^ | RH.A.PRE14.24H | pre |
| M7 | 2^nd^ | RH.A.PRE10.24H + RH.G90.PRE10.12H | pre |
| M8 | 2^nd^ | TRH.15T30nRHG80.POST5.12H + RH.A.POST5.12H + T.A.POST5.24H | post |
| M9 | 2^nd^ | TRH.15T30nRHG80.POST7.12H + RH.A.POST7.12H + T.A.POST7.24H | post |
| M10 | 2^nd^ | TRH.15T30nRHG80.POST10.12H + RH.A.POST10.12H + T.A.4 | post |
| M11 | 2^nd^ | RH7 + T.A.PRE7.24H + T.L9.PRE7.24H | pre |
| M12 | 2^nd^ | TRH.15T30nRHG80.PRE15.24H + T.A.PRE15.24H + T.L9.PRE15.24H | pre |
| M13 | 2^nd^ | RH.A.PRE14.24H + TRH.9T30nRHG90.PRE15.24H + VPD.L45.PRE7.12H | pre |
| M14 | 2^nd^ | RH.G80.PRE14.12H + TRH.9T30nRHG90.PRE15.24H + VPD.L45.PRE7.12H | pre |
| M15 | 2^nd^ | RH7 + T.A.PRE7.24H + sq.T.A.PRE7.24H | pre |
| M16 | 3^rd^ | T.A.5 + RH.G90.CHD.2 + TRH.15T30nRHG80.CHD.3 | post |
| M17 | 3^rd^ | T.A.1 + RH.A.1 + TRH.15T30nRHG80.CHD.1 | pre |
| M18 | 3^rd^ | T.A.3 + RH.G90.CHD.1 + TRH.15T30nRHG80.CHD.2 | pre |
| M19 | 3^rd^ | T.A.2 + RH.A.3 + RH.G90.CHD.3 | pre-to-post |
| M20 | 3^rd^ | T.SD.1 + VPD.A.1 + P.A.3 | pre |
| M21 | 3^rd^ | T.A.2 + TDD.A.2 + P.A.5 | pre |
| M22 | 3^rd^ | T.A.1 + P.A.2 + VPD.L11.CHD.2 | pre |
| M23 | 3^rd^ | P.A.1 + D.A.2 + VPD.L6.CD.1 | pre |
| M24 | 3^rd^ | D.A.1 + TRH.9T30nRHG90.CHD.2 + P.MINMAXDIFF.1 | pre |
| M25 | 3^rd^ | T.A.3 + VPD.L635.CHD.1 + TRH.15T30nRHG90.CHD.1 | pre |
| M26 | 3^rd^ | VPD.A.3 + TRH.15T30nRHG80.CHD.3 | post |
| M27 | 3^rd^ | TRH.15T30nRHG80.CHD.3 + VPD.A.3 + T.A.4 | post |
| M28 | 3^rd^ | VPD.A.4 + T.A.5 + RH.G70.CD.2 | post |
| M29 | 3^rd^ | TDD.SD.3 + RH.G80.CHD.2 + T.G30.CHD.3 | post |
| M30 | 3^rd^ | VPD.SD.3 + TRH.15T30nRHG90.CHD.3 | post |
| M31 | 3^rd^ | TDD.A.4 + T.SD.2 + RH.G90.CHD.2 | post |
| M32 | 3^rd^ | VPD.L6.CD.3 + TRH.5T30nRHG75.CHD.3 | post |
| M33 | 3^rd^ | VPD.A.5 + RH.G70.CD.3 + T.MINMAXDIFF.2 | pre-to-post |
| M34 | 3^rd^ | VPD.A.5 + T.MINMAXDIFF.2 + D.MINMAXDIFF.3 | pre-to-post |
| M35 | 3^rd^ | VPD.L635.CHD.2 + RH.G90.CHD.3 + TDD.A.5 | pre-to-post |
| M36 | 3^rd^ | D.SD.3 + RH.MINMAXDIFF.3 | pre-to-post |
| M37 | 3^rd^ | RH.G70.CD.3 + T.SD.3 | pre-to-post |
| M38 | 3^rd^ | TDD.SD.4 + RH.G80.CHD.3 | pre-to-post |
| M39 | 3^rd^ | VPD.SD.4 + TDD.A.6 | pre-to-post |
