# Supplementary figures and images for: Accuracy in the prediction of disease epidemics when ensembling simple but highly correlated models
Source: PLoS Comput Biol. 2021 Mar 15;17(3):e1008831. doi: 10.1371/journal.pcbi.1008831 (PMC7993824; doi:10.1371/journal.pcbi.1008831)

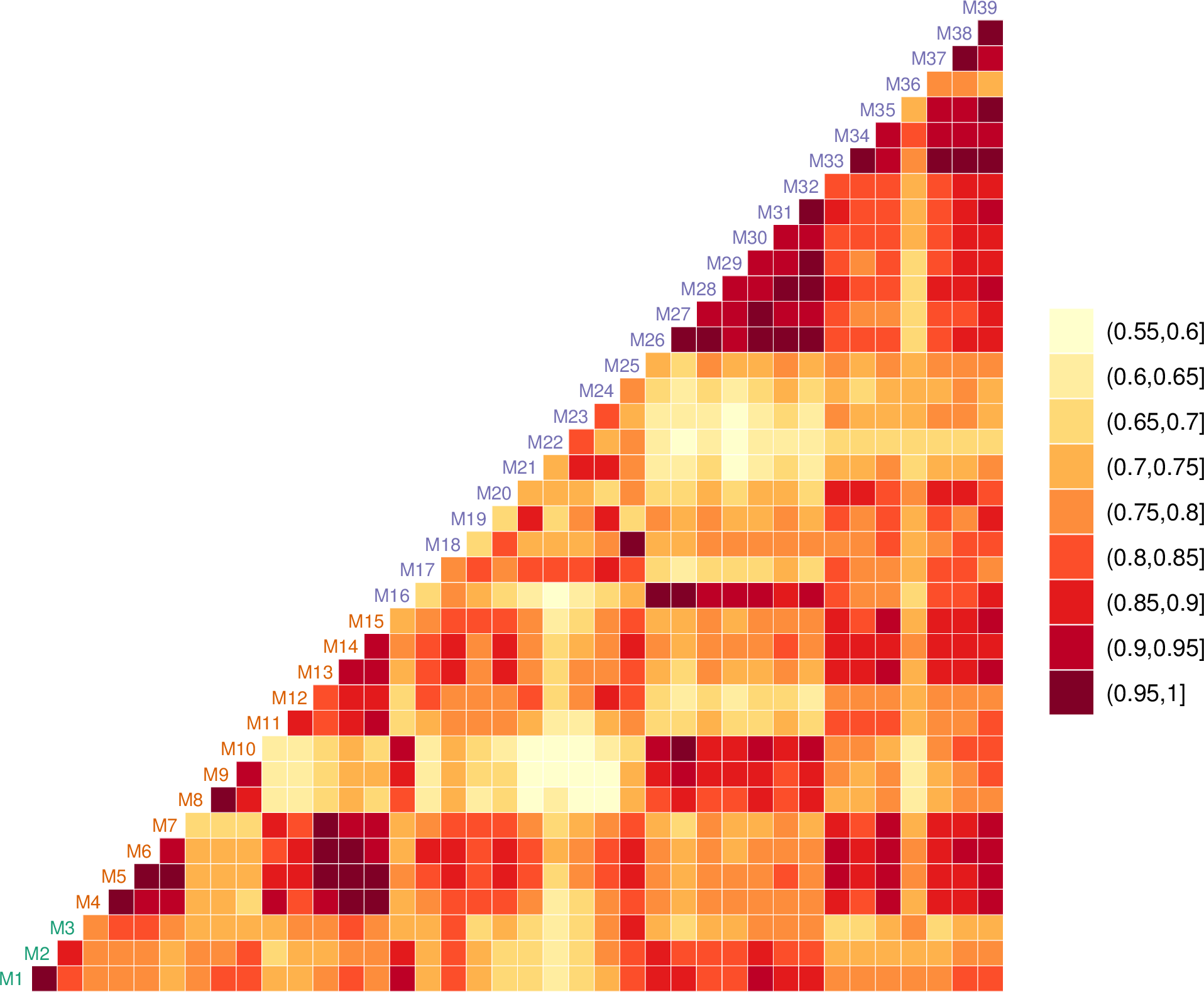

Supplement: S1 Fig — The label colors indicate what generation the model belongs to: green, 1st generation; orange, 2nd generation; purple, 3rd generation. (TIF) [file pcbi.1008831.s003.tif]

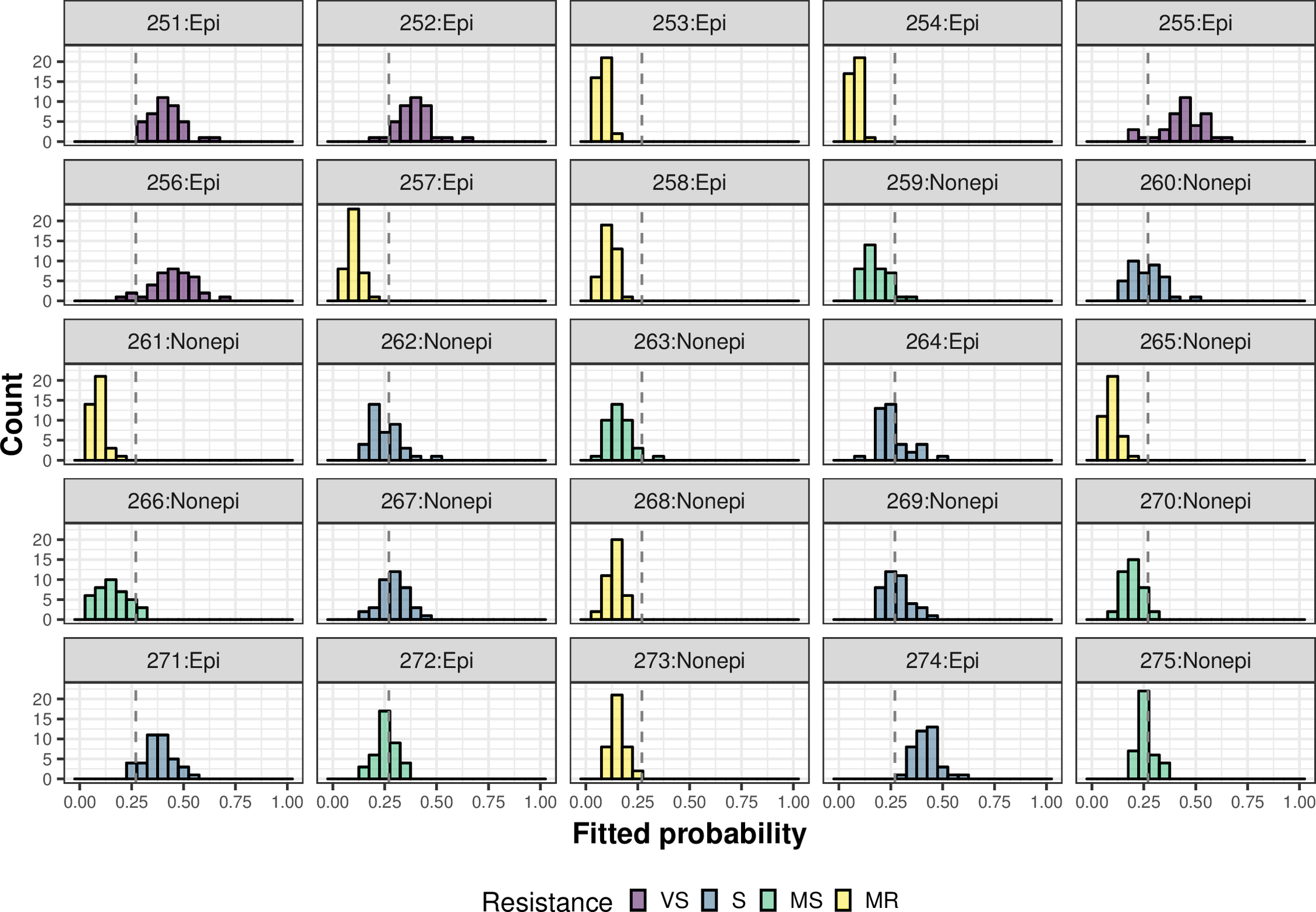

Supplement: S2 Fig — Epi, observation was an epidemic; Nonepi, observation was a non-epidemic. There is a separate panel for each observation. Cultivar resistance levels to Fusarium head blight were VS, very susceptible; S, susceptible; MS, moderately susceptible; MR, moderately resistant. The chosen observations were an arbitrary sample of 25 from the 999 to demonstrate the diversity of distributional results for epidemic predictions. The vertical dashed line in each panel represents the proportion of observations that were FHB epidemics in the data (0.27). (TIF) [file pcbi.1008831.s004.tif]

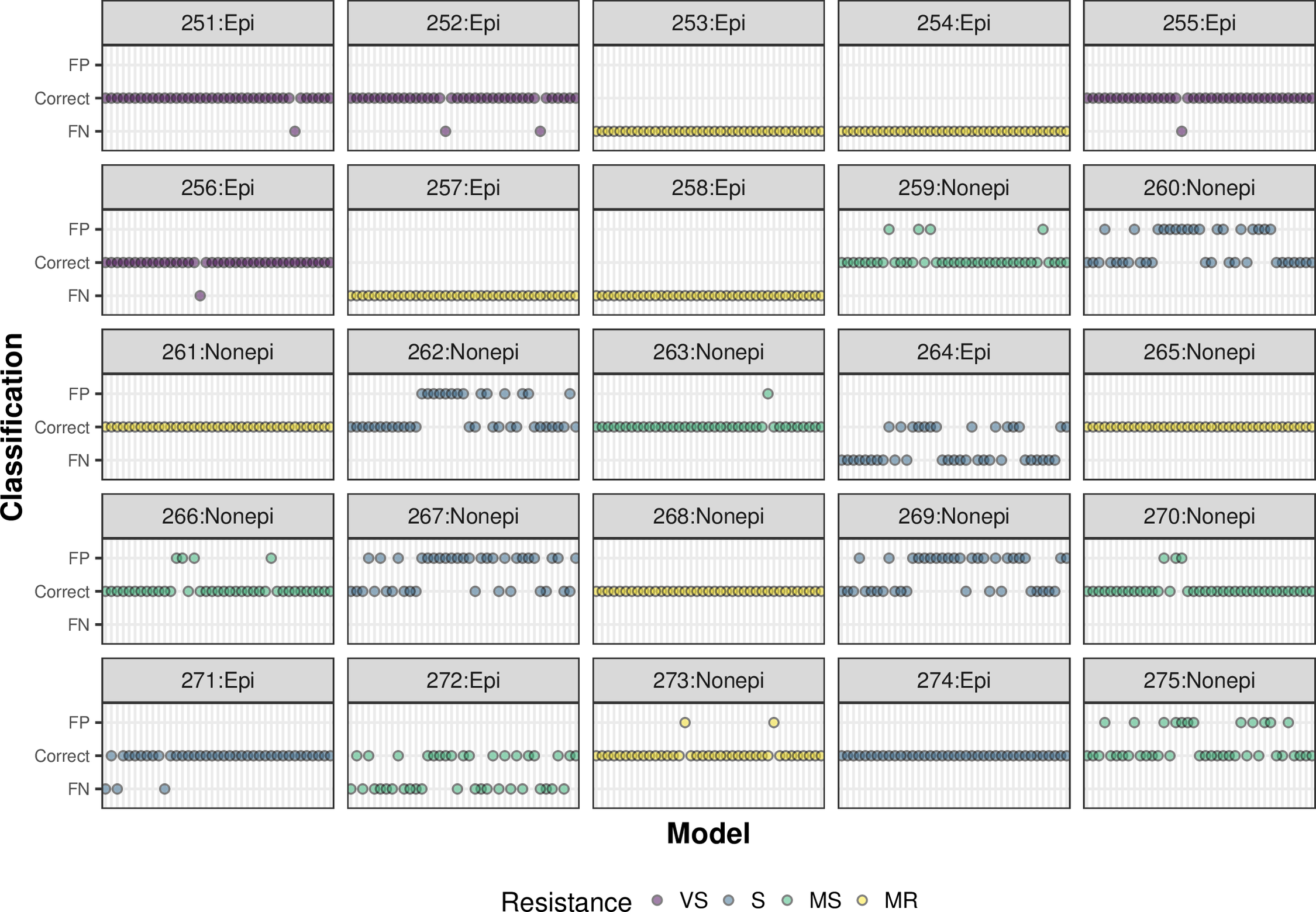

Supplement: S3 Fig — Epi, observation was an epidemic; Nonepi, observation was a non-epidemic. There is a separate panel for each observation. The data points in each panel represent the epidemic classifications by each of the logistic regression models, based on dichotomizing the predicted probability (from cross validation) of an epidemic. For each model the cut-point for classification was that for which the Youden Index was maximal. Correct, observation was correctly classified by the model; FP, observation’s classification was a false positive; FN, observation’s classification was a false negative. Cultivar resistance classes to Fusarium head blight were VS, very susceptible; S, susceptible; MS, moderately susceptible; MR, moderately resistant. The observations are the same arbitrary sample as in S2 Fig to show the diversity of results. (TIF) [file pcbi.1008831.s005.tif]

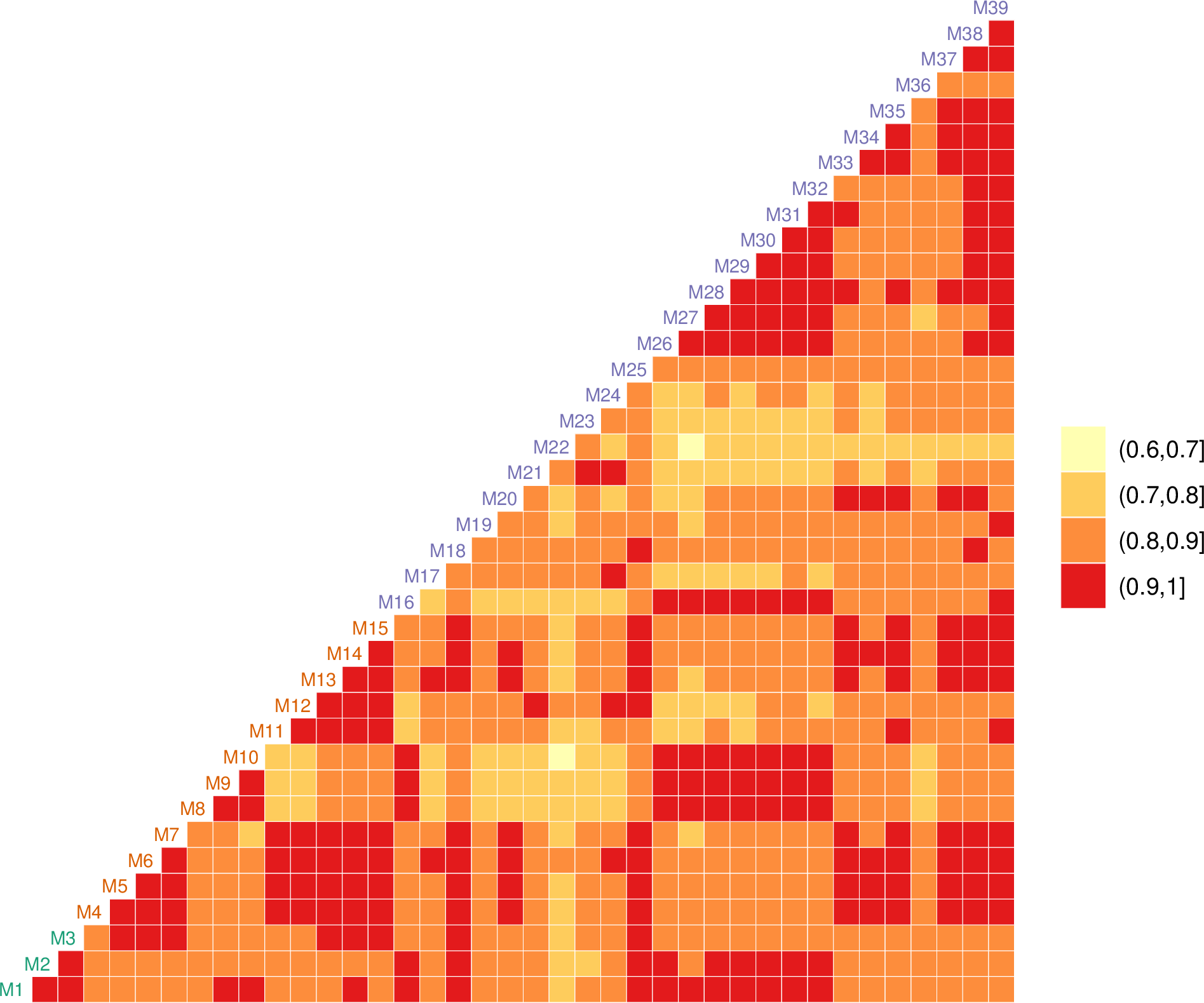

Supplement: S4 Fig — Brier scores were calculated for the predictions of epidemics based on cross-validated fitted probabilities returned by the models for each observation. For each pair of models, the Pearson correlation was calculated between the Brier scores for the 999 observations. The label colors indicate what generation the model belongs to: green, 1st generation; orange, 2nd generation; purple, 3rd generation. (TIF) [file pcbi.1008831.s006.tif]

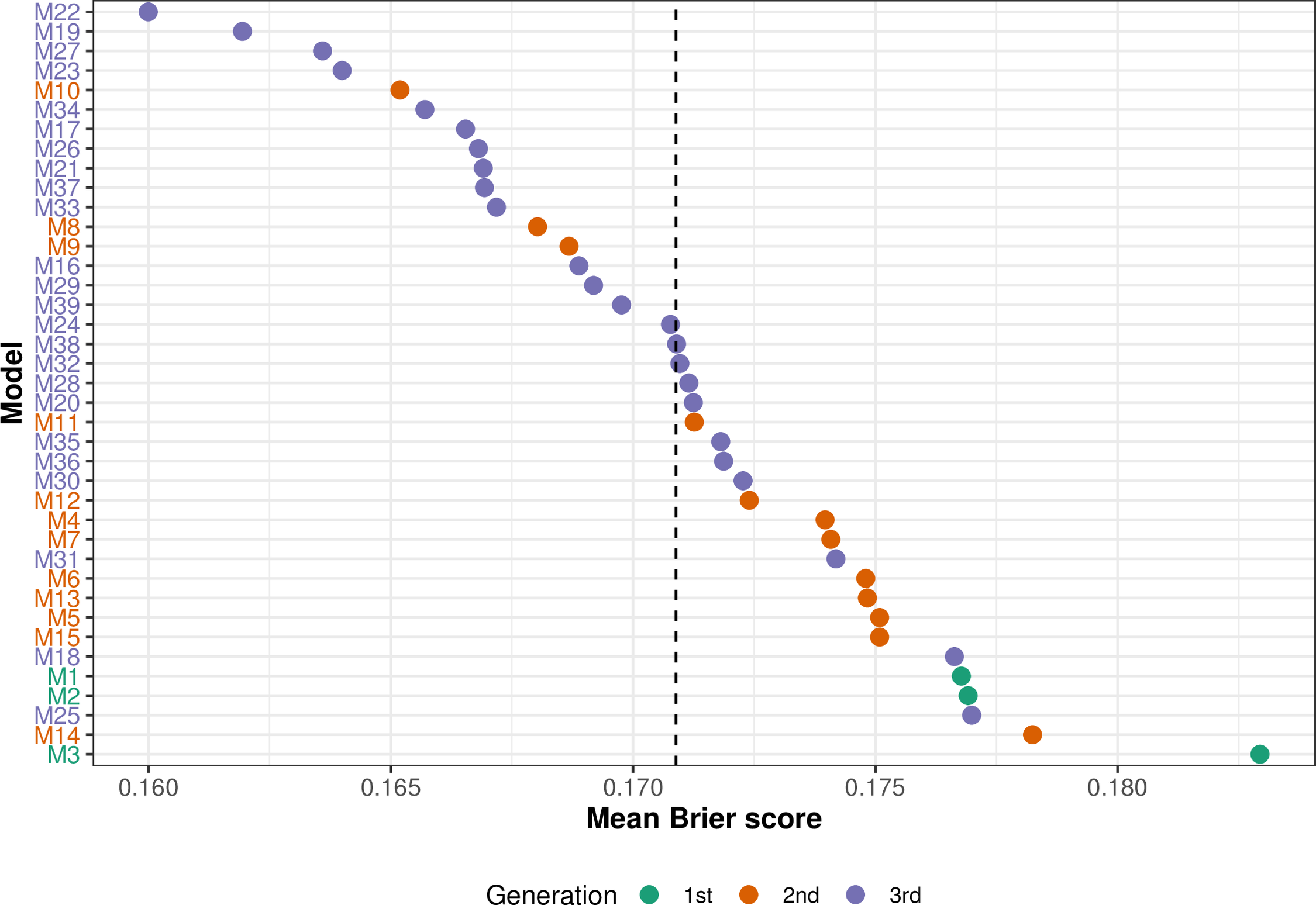

Supplement: S5 Fig — For each of the 39 models, the mean Brier scores were calculated over all observations in the dataset, based on the predicted probabilities of an epidemic (using the cross-validated fitted probabilities). The mean Brier scores are sorted. The dashed line is at the overall mean of 0.171. Mean Brier scores decrease with improving cross-validated fit to the data. The label and point colors indicate what generation the model belongs to: green, 1st generation; orange, 2nd generation; purple, 3rd generation. (TIF) [file pcbi.1008831.s007.tif]

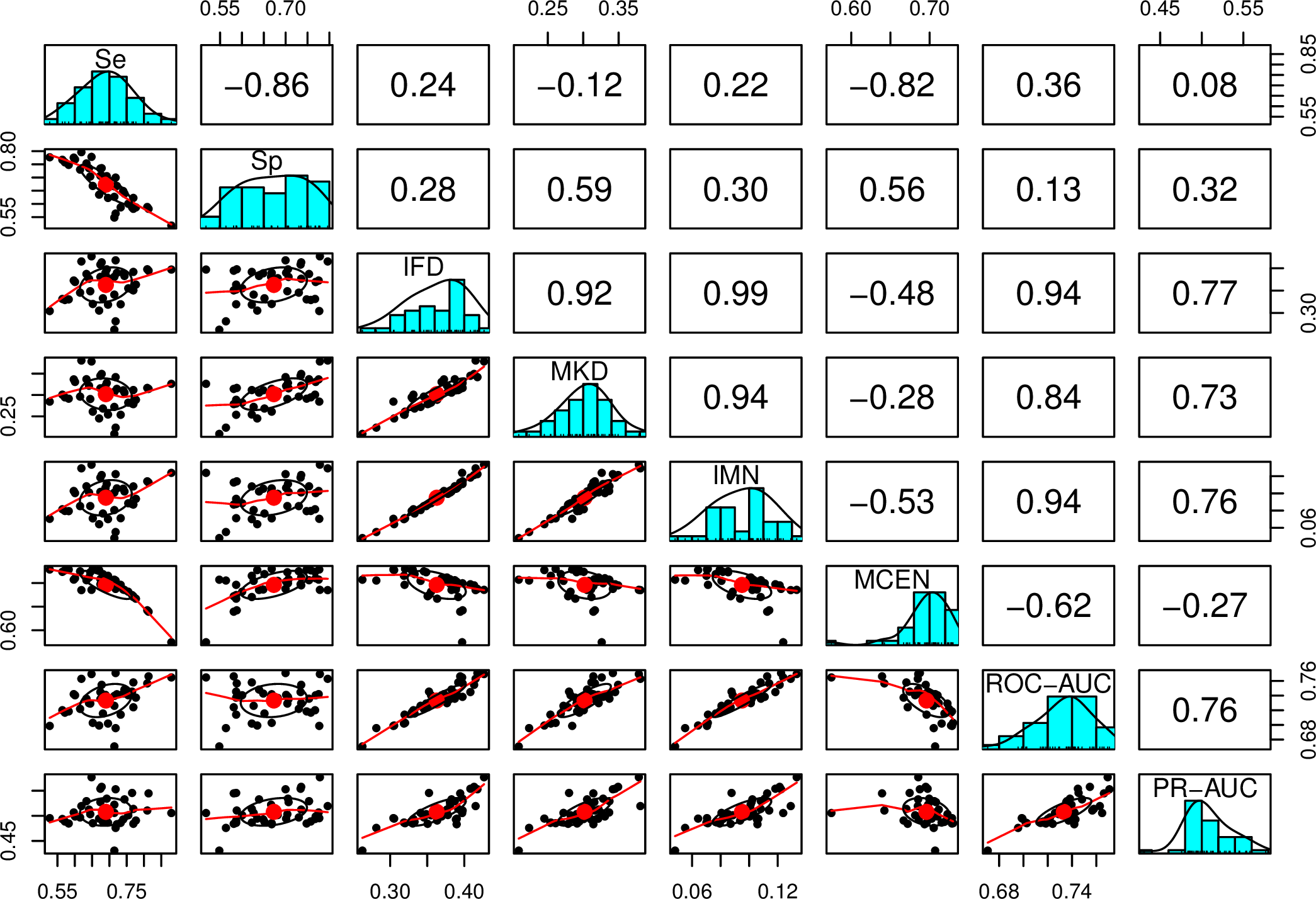

Supplement: S6 Fig — Each graphics panel displays the results for 39 logistic regression models (S2 Table). Metric definitions are in Table 1. (TIF) [file pcbi.1008831.s008.tif]
